# The cardiovascular effects of amodiaquine and structurally related antimalarials: An individual patient data meta-analysis

**DOI:** 10.1371/journal.pmed.1003766

**Published:** 2021-09-07

**Authors:** Xin Hui S. Chan, Ilsa L. Haeusler, Yan Naung Win, James Pike, Borimas Hanboonkunupakarn, Maryam Hanafiah, Sue J. Lee, Abdoulaye Djimdé, Caterina I. Fanello, Jean-René Kiechel, Marcus VG Lacerda, Bernhards Ogutu, Marie A. Onyamboko, André M. Siqueira, Elizabeth A. Ashley, Walter RJ Taylor, Nicholas J. White

**Affiliations:** 1 Mahidol-Oxford Tropical Medicine Research Unit, Faculty of Tropical Medicine, Mahidol University, Bangkok, Thailand; 2 Centre for Tropical Medicine and Global Health, Nuffield Department of Medicine, University of Oxford, Oxford, United Kingdom; 3 University College London Great Ormond Street Institute of Child Health, London, United Kingdom; 4 Health and Diseases Control Unit, Naypyidaw, Myanmar; 5 Department of Clinical Tropical Medicine, Faculty of Tropical Medicine, Mahidol University, Bangkok Thailand; 6 Malaria Research and Training Center, Department of Epidemiology of Parasitic Diseases, Faculty of Pharmacy, University of Science Techniques and Technologies of Bamako, Bamako, Mali; 7 Drug for Neglected Diseases Initiative, Geneva, Switzerland; 8 Fundação de Medicina Tropical Dr Heitor Vieira Dourado, Manaus, Brazil; 9 Instituto Leônidas e Maria Deane (FIOCRUZ-Amazonas), Fundacão Oswaldo Cruz, Manaus, Brazil; 10 Kenya Medical Research Institute, Kisumu, Kenya; 11 Kinshasa School of Public Health, University of Kinshasa, Kinshasa, Democratic Republic of Congo; 12 Instituto Nacional de Infectologia Evandro Chagas, Fundação Oswaldo Cruz, Rio de Janeiro, Brazil; Burnet Institute, AUSTRALIA

## Abstract

**Background:**

Amodiaquine is a 4-aminoquinoline antimalarial similar to chloroquine that is used extensively for the treatment and prevention of malaria. Data on the cardiovascular effects of amodiaquine are scarce, although transient effects on cardiac electrophysiology (electrocardiographic QT interval prolongation and sinus bradycardia) have been observed. We conducted an individual patient data meta-analysis to characterise the cardiovascular effects of amodiaquine and thereby support development of risk minimisation measures to improve the safety of this important antimalarial.

**Methods and findings:**

Studies of amodiaquine for the treatment or prevention of malaria were identified from a systematic review. Heart rates and QT intervals with study-specific heart rate correction (QTcS) were compared within studies and individual patient data pooled for multivariable linear mixed effects regression.

The meta-analysis included 2,681 patients from 4 randomised controlled trials evaluating artemisinin-based combination therapies (ACTs) containing amodiaquine (*n* = 725), lumefantrine (*n* = 499), piperaquine (*n* = 716), and pyronaridine (*n* = 566), as well as monotherapy with chloroquine (*n* = 175) for uncomplicated malaria. Amodiaquine prolonged QTcS (mean = 16.9 ms, 95% CI: 15.0 to 18.8) less than chloroquine (21.9 ms, 18.3 to 25.6, *p* = 0.0069) and piperaquine (19.2 ms, 15.8 to 20.5, *p* = 0.0495), but more than lumefantrine (5.6 ms, 2.9 to 8.2, *p* < 0.001) and pyronaridine (−1.2 ms, −3.6 to +1.3, *p* < 0.001). In individuals aged ≥12 years, amodiaquine reduced heart rate (mean reduction = 15.2 beats per minute [bpm], 95% CI: 13.4 to 17.0) more than piperaquine (10.5 bpm, 7.7 to 13.3, *p* = 0.0013), lumefantrine (9.3 bpm, 6.4 to 12.2, *p* < 0.001), pyronaridine (6.6 bpm, 4.0 to 9.3, *p* < 0.001), and chloroquine (5.9 bpm, 3.2 to 8.5, *p* < 0.001) and was associated with a higher risk of potentially symptomatic sinus bradycardia (≤50 bpm) than lumefantrine (risk difference: 14.8%, 95% CI: 5.4 to 24.3, *p* = 0.0021) and chloroquine (risk difference: 8.0%, 95% CI: 4.0 to 12.0, *p* < 0.001). The effect of amodiaquine on the heart rate of children aged <12 years compared with other antimalarials was not clinically significant. Study limitations include the unavailability of individual patient-level adverse event data for most included participants, but no serious complications were documented.

**Conclusions:**

While caution is advised in the use of amodiaquine in patients aged ≥12 years with concomitant use of heart rate–reducing medications, serious cardiac conduction disorders, or risk factors for torsade de pointes, there have been no serious cardiovascular events reported after amodiaquine in widespread use over 7 decades. Amodiaquine and structurally related antimalarials in the World Health Organization (WHO)-recommended dose regimens alone or in ACTs are safe for the treatment and prevention of malaria.

## Abstract 2

In this meta-analysis, Xin Hui Supanee Chan and colleagues investigate the cardiovascular effects of amodiaquine and structurally-related antimalarials using individual patient data from trials.

## Author summary

**Why was this study done?:**

- Amodiaquine is a widely used antimalarial for the treatment and prevention of malaria.
- The artemisinin-based combination therapy (ACT) artesunate–amodiaquine (ASAQ) is a first-line treatment for uncomplicated malaria in >20 malaria endemic countries.
- Amodiaquine is also the backbone of amodiaquine + sulfadoxine–pyrimethamine, the only drug regimen recommended for seasonal malaria chemoprevention (SMC) given annually to millions of children aged 3 to 59 months across the African Sahel.
- As with other structurally related antimalarials, transient prolongation of the electrocardiographic QT interval and sinus bradycardia has been observed after amodiaquine, but these cardiovascular effects are not well understood.

**What did the researchers do and find?:**

- We conducted secondary analyses, including meta-analyses with multivariable linear mixed effects regression models, of individual patient data from studies of amodiaquine for malaria identified from a systematic review.
- We included data from 2,681 patients from 4 randomised controlled trials evaluating ACTs containing amodiaquine (*n* = 725), lumefantrine (*n* = 499), piperaquine (*n* = 716), and pyronaridine (*n* = 566), as well as monotherapy with chloroquine (*n* = 175) for uncomplicated malaria.
- Amodiaquine prolonged the QT interval assessed with study-specific heart rate correction (QTcS) less than chloroquine and piperaquine, but more than lumefantrine and pyronaridine.
- Amodiaquine was also associated with a higher risk of potentially symptomatic sinus bradycardia compared with other front-line antimalarials in adults and adolescents aged ≥12 years but not in children aged <12 years.

**What do these findings mean?:**

- While caution is advised when using amodiaquine in patients aged ≥12 years who are taking other drugs that reduce heart rate or have serious heart conditions for which heart rate reduction would be undesirable, there have been no serious cardiovascular events reported after extensive use of amodiaquine over 7 decades.
- Our analysis supports the cardiovascular safety of amodiaquine and structurally related antimalarials in the World Health Organization (WHO)-recommended dose regimens alone or in ACTs for the treatment and prevention of malaria.

## Introduction

Malaria remains one of the world’s major causes of preventable death, with more than 200 million cases and over 400,000 people dying of the disease each year, mostly young children in sub-Saharan Africa [1].

Amodiaquine, a 4-aminoquinoline structurally similar to chloroquine, is an antimalarial drug that has been deployed extensively in the treatment and prevention of malaria over the past 60 years. The artemisinin-based combination therapy (ACT) artesunate–amodiaquine (ASAQ) is recommended by the World Health Organization (WHO) for the treatment of uncomplicated *Plasmodium falciparum* and *Plasmodium vivax* malaria in areas without amodiaquine resistance [2]. ASAQ is the first-line oral antimalarial in more than 20 malaria endemic African countries [1]. WHO also recommends that amodiaquine is given together with sulfadoxine–pyrimethamine (AQ + SP) as seasonal malaria chemoprevention (SMC) to children aged 3 to 59 months living in areas of seasonal high-intensity malaria transmission in the Sahel region of Africa. Millions of children are protected by AQ + SP every year [3].

Amodiaquine is generally well tolerated. Life-threatening neutropenia [4] and hepatitis [5] stopped the use of amodiaquine in continuous antimalarial chemoprophylaxis, but there have not been significant adverse effects reported in intermittent prophylaxis. Other serious adverse reactions [6] such as extrapyramidal movement disorders [7,8] are rare [9] following standard regimens for treatment and use in SMC. By contrast, asthenia and asthenia-like reactions have been reported frequently (9% to 36.6%) and have a higher incidence with ASAQ than with other ACTs [8,10,11]. Metabolism of amodiaquine to toxic reactive quinoneimine intermediates [12] may contribute towards development of neutropenia, but the physiological mechanisms underlying its more common adverse drug reactions are not well understood.

The cardiotoxicity of the quinoline and structurally related antimalarials has received renewed interest [13] following the withdrawal of halofantrine because of lethal arrhythmias [13], findings of QT interval prolongation during preregistration safety evaluations of the ACT dihydroartemisinin–piperaquine [14], and recent concerns over the torsadogenic potential of chloroquine and hydroxychloroquine in Coronavirus Disease 2019 (COVID-19) [15]. Chloroquine has potentially lethal cardiovascular toxicity in overdose [13]. However, in the largest safety assessment to date, the risk of sudden unexplained death following dihydroartemisinin–piperaquine at standard malaria treatment doses was not higher than baseline [16]. Data on the cardiovascular effect profile of amodiaquine are scarce despite its widespread use [17]. Amodiaquine was introduced in the early 1950s, when the risk of drug-induced heart rhythm abnormalities was not appreciated.

In the few studies in which patients have undergone electrocardiographic monitoring [18] after amodiaquine treatment, sinus bradycardia [19–21] and QT interval prolongation [19–23] have both been observed. Cardiovascular effects may provide an explanation for the asthenia and asthenia-like reactions commonly reported after amodiaquine. Further characterisation of amodiaquine’s effects on the cardiovascular system could help identify measures to improve safety and tolerability of this important antimalarial. We therefore undertook a meta-analysis of all available clinical data for systematic evaluation of the cardiovascular effects of amodiaquine in comparison to structurally related front-line antimalarials chloroquine, piperaquine, lumefantrine, and pyronaridine, focusing on the heart rate and the electrocardiographic QT interval.

## Methods

### Study selection

Studies were identified from a systematic review of the arrhythmogenic cardiotoxicity of the quinoline and structurally related antimalarials for malaria-related indications, which was last updated on August 21, 2017 [24]. Amodiaquine was one of the 9 antimalarials reviewed. From this review, prospective randomised controlled trials or cohort studies published from 1988 onwards in which 5 or more human participants were given amodiaquine as monotherapy or as part of an ACT in a full 3-day treatment course with systematic electrocardiographic monitoring before and after antimalarial administration were eligible for inclusion in this meta-analysis. Risk of bias of individual studies was assessed using the Pharmacoepidemiological Research on Outcomes of Therapeutics by a European Consortium (PROTECT) [25] checklist for systematic reviews on drug adverse events.

Study authors were contacted with a request for clinical study reports and protocols as well as anonymised individual patient-level datasets of the following prespecified variables identified from expert consultation [13]: age, weight, sex, body temperature, parasitaemia, haemoglobin or haematocrit, blood pressure, heart rate or RR interval duration, uncorrected QT interval duration, QRS interval duration, PR interval duration, ECG abnormalities, other cardiovascular adverse events, antimalarial dose received, concomitant medications, and antimalarial pretreatment received.

All included individual patient-level data were obtained in accordance with appropriate ethical approvals from countries and institutions of origin. Additional ethical approval for this systematic review and meta-analysis of fully anonymised individual patient data was not deemed necessary in keeping with University of Oxford Central University Research Ethics Committee guidance.

### Data extraction and standardisation

Two independent reviewers screened titles, abstracts, full texts, trial documentation, and anonymised datasets and agreed study eligibility. We extracted study-level characteristics including location, patient population, antimalarial treatment indication, antimalarial dosing regimen, and eligibility criteria (Supplementary Methods: Study-Level Data in S1 Appendix) into a standardised database. Where required, trial registry records and investigators were consulted for further information.

Individual patient-level datasets were standardised and checked according to a prespecified data dictionary (Supplementary Methods: Individual Patient-Level Data in S1 Appendix). Only data from scheduled time points and from the first treatment episode for studies of repeated treatments were extracted. Individual patient records were excluded if both QT and RR interval measurements were unavailable or if an antimalarial treatment arm was missing.

### Data analysis

Measurements from fixed and non-fixed dose ASAQ arms were pooled for all analyses as these dose formulations are known to be bioequivalent for amodiaquine and to not have an effect on the pharmacokinetic parameters of these drugs [26,27]. It is generally accepted that artesunate does not have a significant effect on the QT interval [28].

In view of the inverse relationship between the QT interval and heart rate, measured QT intervals were adjusted for heart rate with the widely used Bazett $\left(QTcB=\frac{QT}{\sqrt{RR}}\right)$ and Fridericia ($QTcF=\frac{QT}{\sqrt[{3}]{RR}}$) correction formulae. Age group–specific correction formulae for each study ($QTcS=\frac{QT}{{RR}^{\beta_{age}}}$) were also applied with the correction exponent *β_age_* derived from log–log linear regression (Table A in S1 Appendix). Scatterplots of resulting corrected QT and RR interval relationships with linear regression and 95% CIs were produced for each correction method, individual study, and ECG time point.

All statistical analyses and data visualisation were done in R [29] version 3.6.0, with linear mixed effects modelling conducted using the *nlme* [30] package. Model fit was assessed by visual inspection of residuals, while model discrimination was on the basis of likelihood ratio tests with *p* < 0.05 as the threshold for statistical significance.

#### Study-level analyses

Within each study, electrocardiographic corrected QT interval and heart rate measurements were compared across treatment arms at each available scheduled time point, namely baseline/pre-dose (Day 0), post-dose (Day 2 or 3), and, where available, late (Day 28). Mean corrected QT interval comparisons and categorical analyses of the proportion of participants with corrected QT intervals >450, >480, and >500 milliseconds as well as corrected QT interval prolongation of >30 and >60 milliseconds from baseline at each time point were performed [31]. In addition, mean heart rates as well as the proportion of participants with heart rates of ≤60 and ≤50 beats/minute or age-equivalent thresholds (Table B in S1 Appendix) and their risk differences were evaluated, the higher bradycardia threshold being the lower limit of the normal range and the lower threshold the rate below which individuals could be symptomatic. As body temperature is known to affect both heart rate and the QT interval [24], the median change in body temperature from baseline for each time point was also computed. Means were compared with Welch’s unequal variances *t* test, one-way analysis of variance, or the Kruskal–Wallis test (if the Levene’s test was significant); proportions were compared with Fisher’s exact or the Pearson’s chi-squared test with Yates’ continuity correction, and for medians, distributions were compared with the Wilcoxon rank sum test with continuity correction or the Kruskal–Wallis test as appropriate.

For studies for which cardiovascular vital signs were also available, scatterplots of pulse rate and blood pressure over time with loess regression and 95% CIs were also produced.

#### Individual patient data meta-analyses

Data from baseline/pre-dose (Day 0) and within 6 hours after the final dose of antimalarial treatment (Day 2) from all studies for which these time points were available were pooled for multivariable linear mixed effects analyses. Where there were multiple Day 2 post-dose readings, the recording closest to the time of maximum concentration of the relevant antimalarial was used.

Two sets of models were fitted, with the corrected QT interval and heart rate from ECG measurements as the respective response variables. Study, site, and individual patient were modelled as nested random effects, while fixed effect variable selection was based on directed acyclic graphs of proposed causal relationships among available variables (Figs A–C in S1 Appendix) identified from literature review [24,32] and expert consultation [13]. In the corrected QT interval models, body temperature and antimalarial drug arm as an interaction term with day of ECG measurement were fixed effects. In addition, as age and sex are known to interact to have a nonlinear effect on the QT interval [24], sex was modelled as an interaction term with age group, a categorical variable created from grouping age into 5-year bands. In the heart rate models, body temperature and antimalarial drug arm as an interaction term with day of ECG measurement were again fixed effects. The demographic fixed effect in the heart rate model for adolescents and adults (age ≥12 years) was sex and age in the heart rate model for prepubertal children (age <12 years).

### Reporting

This study is reported as per the Preferred Reporting Items for Systematic Reviews and Meta-Analyses of Individual Patient Data (PRISMA-IPD) guideline (S1 Checklist).

## Results

### Study selection and data availability

**Fig 1:**
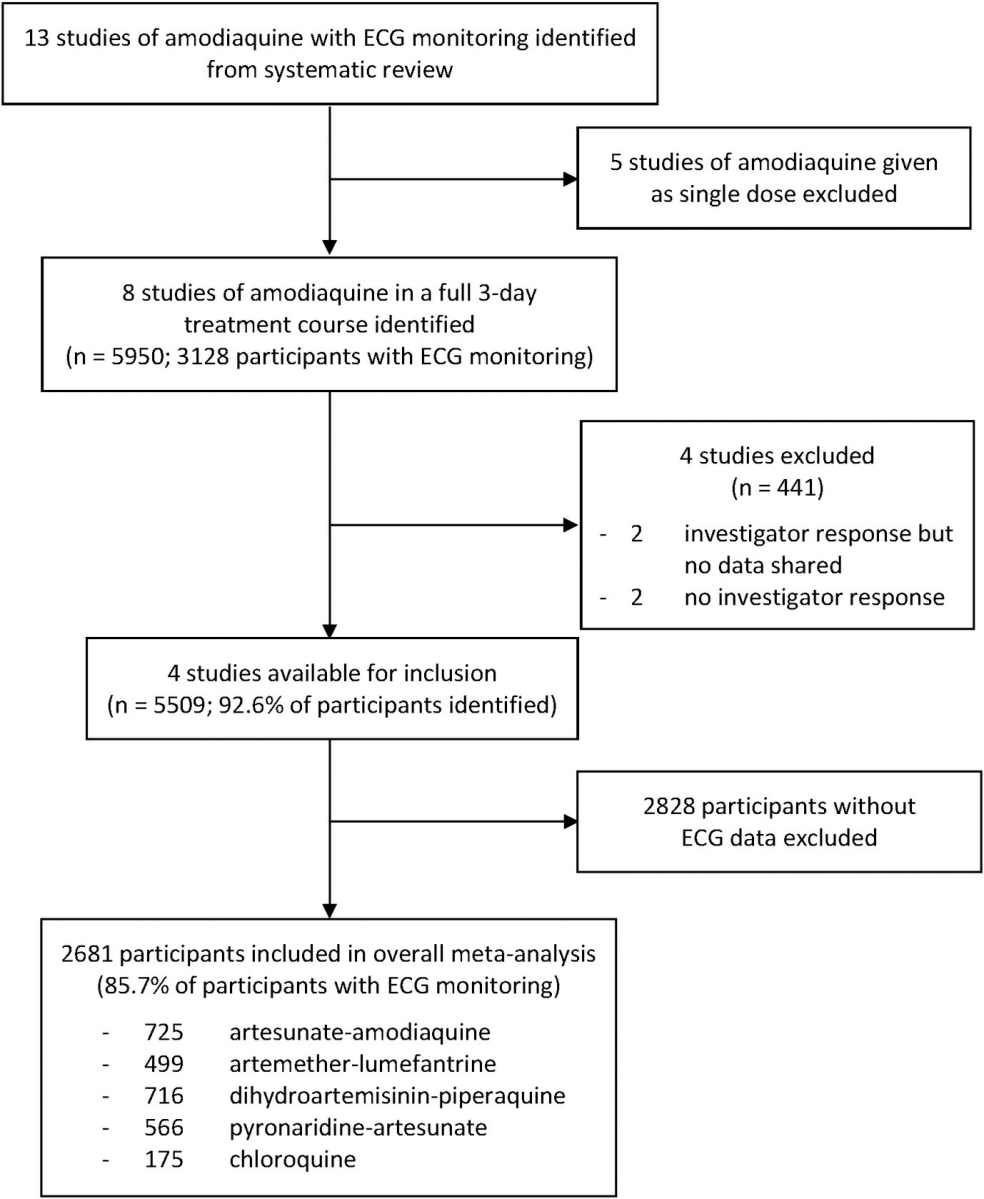
Study selection flowchart.

Our systematic review identified 159 studies of the quinoline and structurally related antimalarials with ECG monitoring before and after antimalarial treatment, of which 13 gave amodiaquine as monotherapy or as an ACT with artesunate. The five [33–37] that were healthy volunteer pharmacokinetic studies in which only a single dose of amodiaquine was administered were excluded. Of the eight [19–23,38–40] eligible studies of amodiaquine in a full 3-day once-daily treatment course for uncomplicated *P*. *falciparum* or *P*. *vivax* malaria, four shared individual patient data and were included (Fig 1). There were no ECG studies of amodiaquine as preventive therapy, i.e., SMC.

There were no cases of sudden cardiac death, life-threatening ventricular tachyarrhythmias (ventricular fibrillation or ventricular tachycardia), torsade de pointes, or any other serious cardiovascular events reported in any of the 5,950 patients in the 8 studies from which individual patient-level data were sought. Of these 5,950 patients, 1,740 were treated with amodiaquine.

The four included studies were randomised controlled trials [21–23,38] comparing the fixed-dose ACT ASAQ with either non-fixed dose amodiaquine with artesunate or another WHO-recommended oral antimalarial in 3-day treatment courses (Fig 1, Table 1, Tables C and D in S1 Appendix). These trials enrolled 5,509 participants, of whom 2,681 had ECG data. These comprised 85.7% of the 3,128 participants identified who had ECG monitoring. All studies measured heart rates and QT intervals, while three [22,23,38] also measured QRS and PR intervals. While all four studies collected cardiovascular vital sign measurements and individual patient-level adverse events, only two [22,23] shared these.

Compared with included studies, the four studies for which data were unavailable were older (all except one completed enrolment before 2007), used amodiaquine in non-fixed dose combinations or monotherapy, had smaller populations from a more restricted demographic, and were less likely to have cardiologists as ECG readers (Table E in S1 Appendix). Other characteristics were otherwise comparable. All studies had low risk of bias overall (Table F in S1 Appendix).

**Table 1:** Characteristics of included studies.

Study reference	Ndiaye and colleagues 2011 [38]	Ogutu and colleagues 2014 [22]	Siqueira and colleagues 2017 [21]	WANECAM 2018 [23]
**Country**	Senegal	Kenya	Brazil	Burkina Faso (2 sites), Guinea (1 site), Mali (3 sites)
**Trial population (with ECG monitoring)**	Adults and children (ECG monitoring if age ≥12 years)	Adults (all)	Adults and children (ECG monitoring if age >10 years)	Adults and children (all)
**Treatment indication**	Uncomplicated *P*. *falciparum* malaria	Uncomplicated *P*. *falciparum* malaria	Uncomplicated *P*. *vivax* malaria	Uncomplicated *P*. *falciparum* malaria
**Days of follow-up**	28	28	42	42
**Amodiaquine-containing drugs (manufacturer)**	ASAQ (Sanofi)	ASAQ (Sanofi) and AQ + AS (Sanofi + Guilin)	ASAQ (Sanofi)	ASAQ (Sanofi)
**Non-amodiaquine drugs (manufacturer)**	AL (Novartis)	None	CQ (Farmanguinhos)	AL (Novartis), DP (Sigma Tau), PA (Shin Poong)
**Directly observed therapy**	All doses	All doses	All doses	All doses
**Number of participants in amodiaquine drug arms (with ECG monitoring)**	184 (77)	54 (54)	189 (179)	1,061 (417)
**Number of participants in non-amodiaquine drug arms (with ECG monitoring)**	182 (77)	0	190 (175)	3,649 (1,708)
**TdP risk factors excluded at screening**	Yes	Yes	No	Yes
**PK sampling (food intake)**	No	Yes (starved)	No	No
**Vital sign measurement time points, protocol days**	0 (pre-dose), 1, 2, 3, 7, 14, 28	0 (pre-dose), 1, 2, 3, 7, 14, 21, 28	0 (pre-dose), 1, 2, 3, 7, 14, 28, 42	0 (pre-dose), 1, 2, 3, 7, 14, 28, 35, 42
**ECG measurement time points, protocol days**	0 (pre-dose), 3 (post-dose)	0 (pre-dose, +2 hours, +4 hours), 2 (+2 hours, +4 hours), 28	0 (pre-dose), 2 (post-dose), 28	0 (pre-dose), 2 (+2 to +4 hours)
**PK sampling time points, protocol days**	None	0 (pre-dose, +0.25 to +4 hours), 1 (+0.25 to +4 hours), 2 (+0.25 to +4 hours), 7, 14, 21, 28	None	None

AL, artemether–lumefantrine fixed-dose combination; AQ + AS, artesunate + amodiaquine non-fixed dose combination; ASAQ, artesunate–amodiaquine fixed-dose combination; CQ, chloroquine; DP, dihydroartemisinin–piperaquine fixed-dose combination; ECG, electrocardiogram; PA, pyronaridine–artesunate fixed-dose combination; PK, pharmacokinetic; TdP, torsades de pointes; WANECAM, West African Network for Clinical Trials of Antimalarial Drugs.

### Baseline and dosing characteristics of ECG population

Baseline characteristics of the population included in the ECG interval analyses are summarised in Table 2. The multisite West African Network for Clinical Trials of Antimalarial Drugs (WANECAM) 2018 study (*n* = 2,125) had the youngest ECG population with a median age of 8.3 years (IQR: 5.1 to 12.1, range: 0.5 to 71.6), followed by the Ndiaye and colleagues 2011 study from Senegal (*n* = 148) with a median age of 15.0 years (IQR: 13.0 to 20.0, range: 11.0 to 65.0) and the Ogutu and colleagues 2014 study from Kenya (*n* = 54) with a median age of 24.0 years (IQR: 19.0 to 32.0, range: 18.0 to 60.0). These three uncomplicated *P*. *falciparum* studies from sub-Saharan Africa had otherwise clinically comparable baseline disease characteristics of temperature and parasitemia between treatment arms and across studies. The remaining study, Siqueira and colleagues 2017 from Brazil (*n* = 354), was of older and mostly male (75.1%, 266/354) adults with *P*. *vivax* malaria who had a median age of 36.4 years (IQR: 26.6 to 48.7, range: 10.3 to 74.9).

**Table 2:** Baseline characteristics of included population in ECG interval analysis.

Study reference	Ndiaye and colleagues 2011 [38]		Ogutu and colleagues 2014 [22]	Siqueira and colleagues 2017 [21]		WANECAM 2018 [23]			
**Drug arm**	*ASAQ*	*AL*	*ASAQ*	*ASAQ*	*CQ*	*ASAQ*	*AL*	*DP*	*PA*
Number of Individuals	75	73	54	179	175	417	426	716	566
**Age (years)**									
Median (IQR)	19.7 (13.0 to 18.0)	16.0 (14.0 to 20.0)	24.0 (19.0 to 32.0)	37.4 (25.1 to 50.9)	35.8 (28.5 to 47.7)	7.7 (5.0 to 11.4)	8.6 (5.0 to 13.2)	8.1 (5.1 to 11.3)	9.0 (5.5 to 13.6)
<12	1 (1.3%)	1 (1.4%)	0	2 (1.1%)	1 (0.6%)	335 (80.3%)	298 (70.0%)	567 (79.2%)	375 (66.3%)
0.5 to <2	0	0	0	0	0	11 (2.6%)	10 (2.3%)	18 (2.5%)	7 (1.2%)
2 to <5	0	0	0	0	0	95 (22.8%)	90 (21.1%)	150 (20.9%)	99 (17.5%)
5 to <12	1 (1.3%)	1 (1.4%)	0	2 (1.1%)	1 (0.6%)	229 (54.9%)	198 (46.5%)	399 (55.7%)	269 (47.5%)
≥12	74 (98.7%)	72 (98.6%)	54 (100%)	177 (98.9%)	174 (99.4%)	82 (19.7%)	128 (30.0%)	149 (20.8%)	191 (33.7%)
12 to <18	52 (69.3%)	45 (61.6%)	0	18 (10.0%)	14 (8.0%)	71 (17.0%)	97 (22.8%)	116 (16.2%)	122 (21.6%)
18 to <35	9 (12.0%)	21 (28.8%)	43 (79.6%)	62 (34.6%)	64 (36.6%)	8 (1.9%)	22 (5.2%)	25 (3.5%)	53 (9.4%)
35 to <50	11 (14.7%)	4 (5.5%)	8 (14.8%)	50 (28.2%)	62 (35.4%)	3 (0.7%)	6 (1.4%)	6 (0.8%)	14 (2.5%)
≥50	2 (2.7%)	2 (2.7%)	3 (5.6%)	47 (26.3%)	34 (19.4%)	0	3 (0.7%)	2 (0.3%)	2 (0.4%)
**Weight (kg)**									
Median (IQR)	40.1 (33.3 to 52.8)	48.6 (32.4 to 56.8)	59.0 (53.0 to 62.0)	71.0 (61.1 to 80.5)	72.0 (61.7 to 82.0)	20.7 (15.9 to 28.2)	22.5 (16.2 to 34.2)	22.0 (16.1 to 30.7)	24.3 (17.2 to 41.6)
**Sex**									
Female	33 (44.0%)	32 (43.8%)	29 (53.7%)	46 (25.7%)	42 (24.0%)	190 (45.6%)	208 (48.8%)	348 (48.6%)	291 (51.4%)
Male	42 (56.0%)	41 (56.2%)	25 (46.3%)	133 (74.3%)	133 (76.0%)	227 (54.4%)	218 (51.2%)	368 (51.4%)	275 (48.6%)
**Temperature (°C)**									
Median (IQR)	37.5 (37.0 to 38.8)	37.7 (36.7 to 39.0)	37.7 (37.1 to 38.6)	37.7 (36.7 to 38.7)	37.4 (36.7 to 38.5)	38.1 (37.3 to 38.9)	38.2 (37.4 to 39.1)	38.2 (37.3 to 39.0)	38.2 (37.3 to 39.1)
≥37.5	39 (52.0%)	39 (53.4%)	35 (64.8%)	104 (58.1%)	87 (49.7%)	298 (71.5%)	311 (73.0%)	515 (71.9%)	402 (71.0%)
**Parasitaemia (parasites/μL)**									
Median (IQR)	12,677 (4,835 to 40,263)	15,813 (5,537 to 32,045)	17,181 (5,317 to 25,876)	1,978 (836 to 3,739)	1,764 (819 to 3,373)	12,940 (840 to 37,160)	24,980 (4,380 to 67,530)	15,680 (1,280 to 41,640)	18,170 (1,695 to 51,870)
>10,000 to 50,000	29 (38.7%)	33 (45.2%)	24 (44.4%)	15 (8.4%)	4 (2.3%)	153 (36.7%)	150 (35.2%)	269 (37.6%)	195 (34.5%)
>50,000 to 100,000	9 (12.0%)	12 (16.4%)	6 (11.1%)	0	0	45 (10.8%)	71 (16.7%)	84 (11.7%)	92 (16.3%)
>100,000 to 250,000	6 (8.0%)	0	1 (1.9%)	0	0	29 (7.0%))	64 (15.0%)	54 (7.5%)	60 (10.6%)
**Haemoglobin (g/dL)**									
Median (IQR)	12.5 (11.7 to 13.7)	12.1 (11.1 to 13.6)	13.2 (11.9 to 14.8)	13.5 (12.4 to 14.8)	13.6 (12.5 to 14.5)	10.5 (9.6 to 11.3)	10.7 (9.7 to 11.6)	10.6 (9.6 to 11.5)	10.8 (9.8 to 11.8)
<8	0	1 (1.4%)	0	0	1 (0.6%)	17 (4.1%)	15 (3.5%)	41 (5.7%)	26 (4.6%)
**Heart rate (bpm)**									
Mean (SD)	94.9 (19.2)	95.8 (21.9)	91.7 (15.7)^a^	90.3 (21.3)	88.4 (19.1)	109.1 (22.0)	108.8 (22.0)	108.9 (21.6)	106.0 (22.8)
≥140	1 (1.3%)	2 (2.7%)	0	2 (1.1%)^b^	1 (0.6%)^b^	37 (8.9%)	36 (8.5%)	57 (8.0%)	45 (8.0%)
120 to <140	5 (6.7%)	10 (13.7%)	2 (3.8%)^a^	17 (9.6%)	6 (3.5%)^b^	99 (23.7%)	96 (22.5%)	169 (23.6%)	119 (21.0%)
100 to <120	27 (36.0%)	15 (20.5%)	17 (32.1%)^a^	42 (23.7%)^b^	39 (22.3%)^b^	138 (33.1%)	149 (35.0%)	248 (34.6%)	174 (30.7%)
80 to <100	23 (30.7%)	26 (35.6%)	21 (39.6%)^a^	56 (31.6%)^b^	69 (39.9%)^b^	110 (26.4%)	105 (24.6%)	177 (24.7%)	154 (27.2%)
60 to <80	17 (22.7%)	18 (24.7%)	13 (24.5%)^a^	51 (28.8%)^b^	49 (28.3%)^b^	29 (7.0%)	38 (8.9%)	57 (8.0%)	70 (12.4%)
<60	2 (2.7%)	2 (2.7%)	0	9 (5.1%)^b^	9 (5.2%)^b^	4 (1.0%)	2 (0.5%)	8 (1.1%)	4 (0.7%)

^a^ One participant had a missing baseline heart rate.

^b^ Two participants had missing baseline heart rates.

AL, artemether–lumefantrine; ASAQ, artesunate–amodiaquine; bpm, beats per minute; CQ, chloroquine; DP, dihydroartemisinin–piperaquine; ECG, electrocardiogram; IQR, interquartile range; PA, pyronaridine–artesunate; SD, standard deviation.

Most of the 725 participants treated with ASAQ received total mg/kg doses of amodiaquine over 3 days within the WHO-recommended range of 22.5 to 45 mg/kg body weight, other than the 86 (11.9%) adult participants weighing >72kg who received doses <22.5 mg/kg (Figs D and E in S1 Appendix). A total of 147 (84%) of the 175 participants treated with chloroquine, 139 (94%) of whom were adults weighing >60 kg, received total chloroquine doses below WHO-recommended dose of 25 mg/kg body weight. Of the 716 participants treated with dihydroartemisinin–piperaquine, 177 (24.7%) received a total dose of piperaquine below WHO-recommended range of 48 to 81 mg/kg, 161 (91%) of whom were children weighing <25 kg. All participants treated with artemether–lumefantrine and pyronaridine–artesunate received total doses of lumefantrine and pyronaridine within WHO-recommended ranges of 29 to 144 and 22.5 to 45 mg/kg body weight, respectively (Fig F in S1 Appendix).

### Corrected QT interval—Study-level analyses

Individual study-level analyses of corrected QT interval measurements are presented in Table G in S1 Appendix.

**Fig 2:**
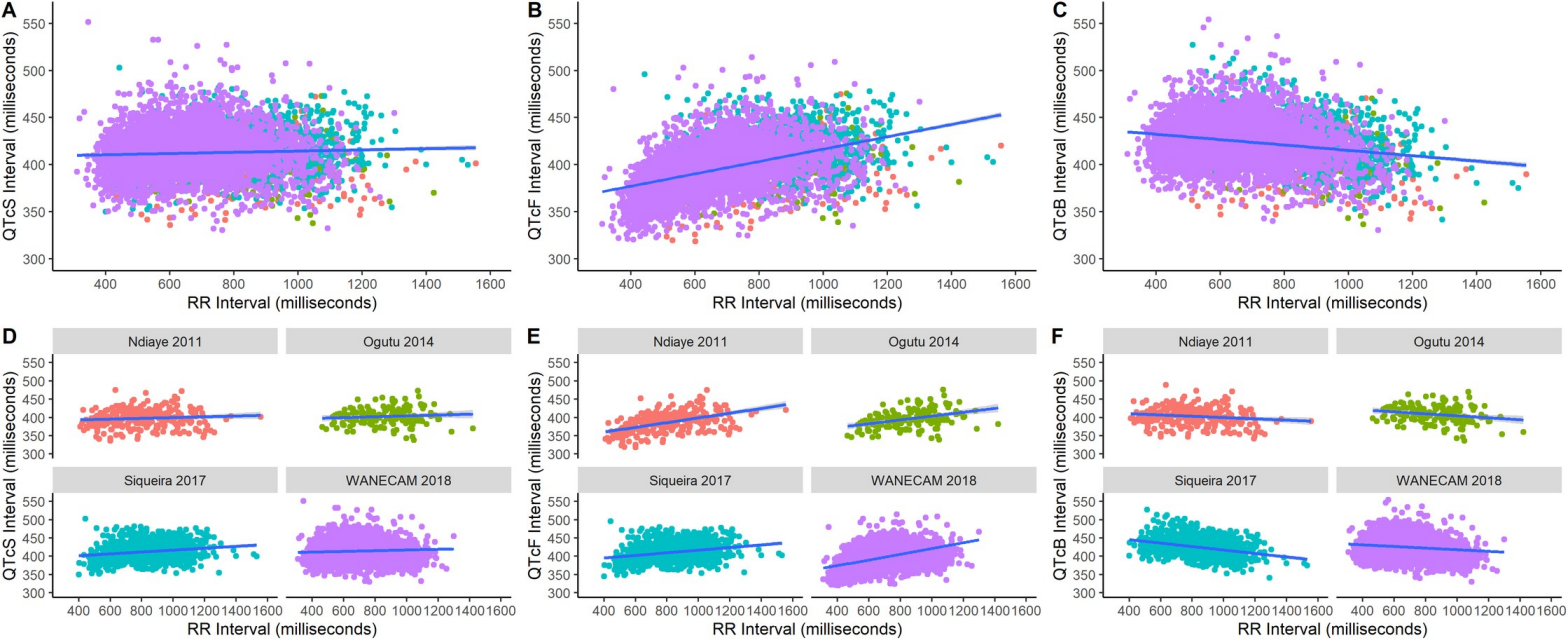
Corrected QT interval and RR interval relationships by correction method and individual study. QT intervals adjusted with study-specific ($QTcS=\frac{QT}{{RR}^{\beta_{age}}}$, where *β_age_* decreased with increasing age), Fridericia ($QTcF=\frac{QT}{\sqrt[{3}]{RR}}$), and Bazett heart rate corrections ($QTcB=\frac{QT}{\sqrt{RR}}$) and their relationship with RR intervals in the pooled dataset (top panels) and by individual study (bottom panels) with means (blue line) and 95% CIs (shaded area) from linear regression. A slight positive relationship is expected even with ideal correction because of confounding by body temperature changes, i.e., fever and defervescence, in malaria.

The study-specific correction provided the least heart rate dependence of the QT interval for all individual studies and the pooled dataset (Fig 2, Fig G in S1 Appendix). In the International Council for Harmonisation of Technical Requirements for Pharmaceuticals for Human Use (ICH)-recommended categorical analyses, the use of the Bazett heart rate correction overestimated the numbers of individuals with values beyond absolute thresholds of corrected QT interval prolongation (>450, >480, and >500 milliseconds), while the use of the Fridericia heart rate correction underestimated these. For change from baseline thresholds (>30 and >60 milliseconds), the use of the Bazett heart rate correction underestimated, while the use of the Fridericia heart rate correction overestimated the number of individuals with corrected QT interval prolongation as determined by change from baseline (Table G in S1 Appendix).

### Corrected QT interval—Individual patient data meta-analysis

**Fig 3:**
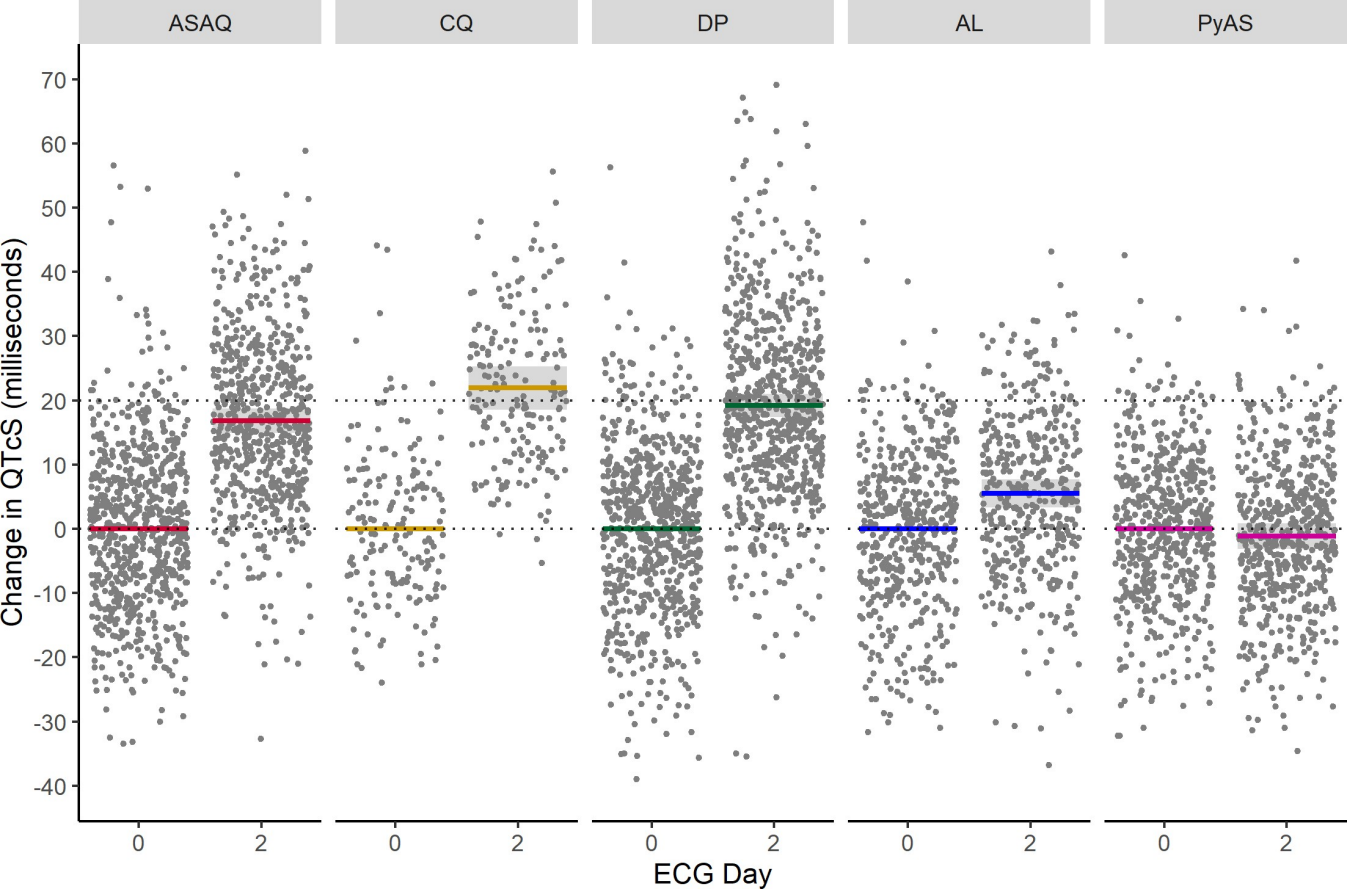
Change in corrected QT interval after antimalarial treatment for uncomplicated malaria. Change in QT interval adjusted with study-specific heart rate correction (QTcS) after treatment with the antimalarials artesunate-amodiaquine (ASAQ), chloroquine (CQ), dihydroartemisinin-piperaquine (DP), artemether-lumefantrine (AL), and pyronaridine-artesunate (PyAS) from a multivariable linear mixed effects regression model adjusting for age group, sex, body temperature, and individual study/site/person effects. Lines indicate means with colour representing antimalarial treatment arm, shaded areas denote 95% confidence intervals, and dots are partial residuals.

**Fig 4:**
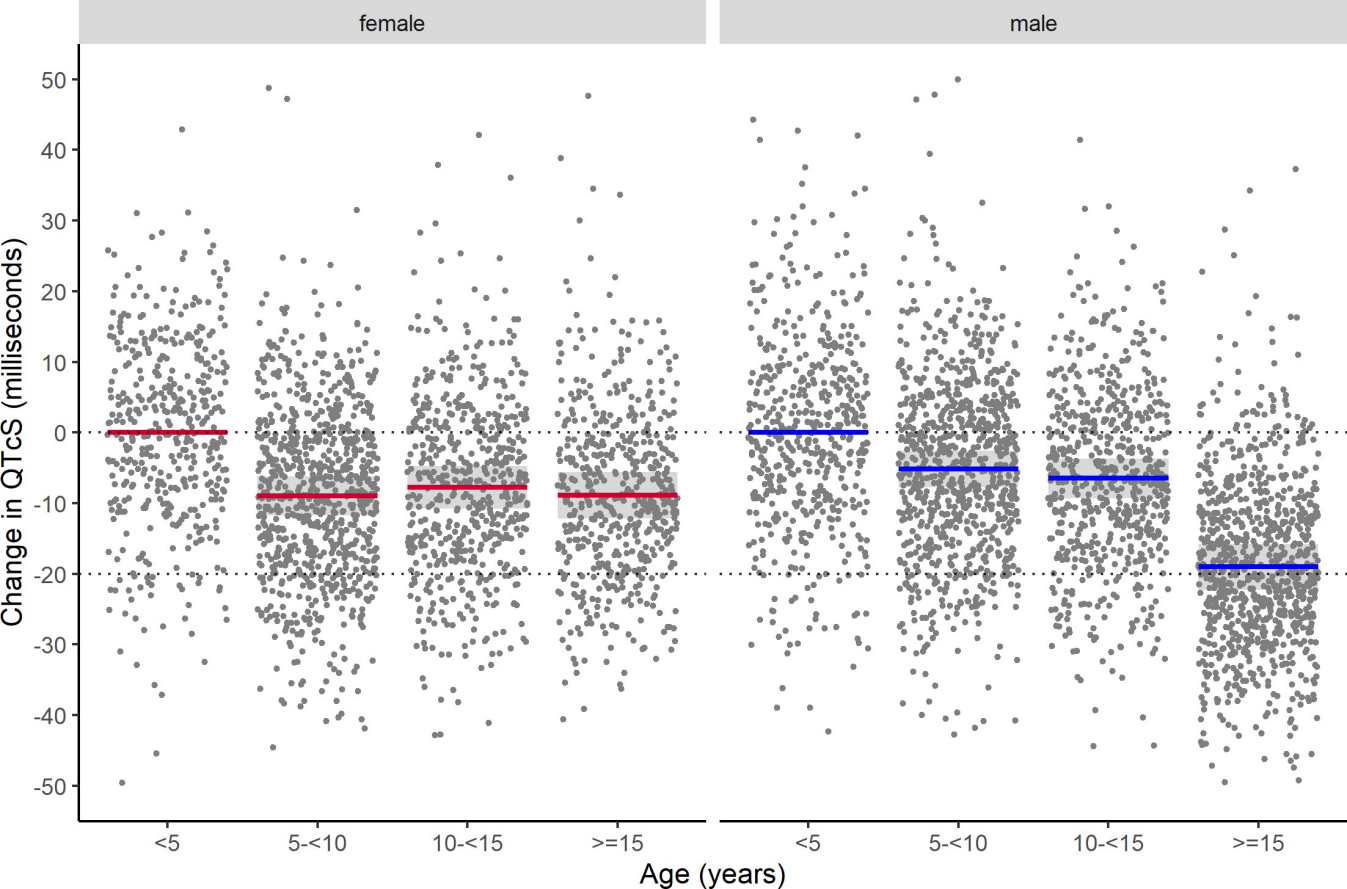
Differences in corrected QT interval by age group and sex. Mean differences in QT interval adjusted with study-specific heart rate correction (QTcS) by age group and sex compared with the mean value for children aged <5 years from a multivariable linear mixed effects regression model adjusting for treatment day, antimalarial treatment arm, body temperature, and individual study/site/person effects. Lines indicate means with colour representing sex, shaded areas denote 95% confidence intervals, and dots are partial residuals.

QT interval data were available for 2,681 patients. Results from multivariable linear mixed effects modelling for the QT interval with study-specific heart rate correction (QTcS) are summarised in Table 3. Chloroquine was associated with the greatest mean increase in QTcS from baseline to day 2 (21.9 milliseconds, 95% CI: 18.3 to 25.6), followed by dihydroartemisinin–piperaquine (19.2 milliseconds, 95% CI: 15.8 to 20.5), ASAQ (16.9 milliseconds, 95% CI: 15.0 to 18.8), artemether–lumefantrine (5.6 milliseconds, 95% CI: 2.9 to 8.2), and pyronaridine–artesunate (−1.2 milliseconds, 95% CI: −3.6 to 1.3) (Fig 3). The QTcS prolongation after ASAQ was less than that after chloroquine (*p* = 0.0069) and dihydroartemisinin–piperaquine (*p* = 0.0495) and more than that after artemether–lumefantrine (*p* < 0.001) and pyronaridine–artesunate (*p* < 0.001). Body temperature was associated with a mean increase in QTcS of 2.7 milliseconds (95% CI: 2.2 to 3.3) per 1°C of defervescence with all other variables held constant. QTcS became shorter after infancy and early childhood and was comparable in both sexes until around puberty (10 to <15 years) when QTcS shortened in males (10.1 milliseconds, 95% CI: 6.0 to 14.0) but not females (Fig 4). The differences in QTcS between ASAQ and comparator antimalarials at baseline were neither clinically nor statistically significant after adjustment for body temperature, age group, and sex.

**Table 3:** Multivariable linear mixed effects analysis of QT interval with study-specific correction.

Variable	Number of observations	Adjusted estimate (95% CI)	*p*-value
Change from baseline after ASAQ	1,361	16.87 (14.97 to 18.78) milliseconds	<0.001
Change from baseline versus ASAQ, by antimalarial treatment arm	5,192		
ASAQ	1,361	Reference	
CQ	347	5.07 (1.39 to 8.75) milliseconds	0.0069
DP	1,429	2.34 (0.01 to 4.67) milliseconds	0.0495
AL	924	−11.32 (−13.96 to −8.67) milliseconds	<0.001
PA	1,131	−18.03 (−20.51 to −15.56) milliseconds	<0.001
Baseline measurement versus ASAQ, by antimalarial treatment arm	2,674		
ASAQ	720	Reference	
CQ	173	−1.46 (−5.43 to 2.50) milliseconds	0.4693
DP	716	1.29 (−1.07 to 3.64) milliseconds	0.2833
AL	499	−0.33 (−3.04 to 2.37) milliseconds	0.8091
PA	566	−0.92 (−3.49 to 1.64) milliseconds	0.4808
Body temperature, per 1°C increase	5,192	−2.74 (−3.33 to −2.15) milliseconds	<0.001
Female sex, by age group	1,215		
0.5 to <5 years	215	Reference	
5 to <10 years	419	−8.96 (−11.75 to −6.17) milliseconds	<0.001
10 to <15 years	294	−7.77 (−10.81 to −4.74) milliseconds	<0.001
≥15 years	287	−8.89 (−12.17 to −5.61) milliseconds	<0.001
Male versus female sex, by age group	1,459		
0.5 to <5 years*	265	−3.14 (−6.19 to −0.10) milliseconds	0.0431
5 to <10 years*	413	3.79 (−0.02 to 7.61) milliseconds	0.0514
10 to <15 years*	329	1.28 (−2.79 to 5.36) milliseconds	0.5381
≥15 years*	452	−10.05 (−14.09 to −6.00) milliseconds	<0.001

* Coefficient to be added to female coefficient of same age group.

AL, artemether–lumefantrine; ASAQ, artesunate–amodiaquine; CQ, chloroquine; DP, dihydroartemisinin–piperaquine; PA, pyronaridine–artesunate.

### Heart rate—Study-level analyses

Individual study-level analyses of ECG heart rate measurements are presented in Table H in S1 Appendix.

A total of 56% (171/305) of participants who received ASAQ developed sinus bradycardia of ≤60 beats/minute and 9.5% (29/305) had a bradycardia of ≤50 beats/minute on day 2 or 3 (post-dose) in each of the three studies with median ages of ≥12 years. Of these studies, two had comparator arms: patients treated with ASAQ had a higher relative risk of sinus bradycardia than those treated with artemether–lumefantrine (≤60 beats/minute risk difference: 33.4%, 95% CI: 18.2 to 48.6, *p* < 0.001; ≤50 beats/minute: 14.8, 95% CI: 5.4 to 24.3, *p* = 0.0021) and chloroquine (≤60 beats/minute risk difference: 33.6%, 95% CI: 24.0 to 43.1, *p* < 0.001; ≤50 beats/minute risk difference: 8.0, 95% CI: 4.0 to 12.0, *p* < 0.001). In the remaining study with a median age <12 years, the absolute and relative risks of developing sinus bradycardia within and between treatment arms were not clinically significant.

### Pulse rate and blood pressure—Study-level analyses

Cardiovascular vital sign measurements from each study visit were available from 2 studies (Figs H and I in S1 Appendix). Consistent with the ECG interval data, mean pulse rates fell from day 0 to 3 with lower pulse rates in the bradycardic range (≤60 beats/minute) after ASAQ compared with chloroquine before returning to similar levels from day 7 for the remaining 3 to 5 weeks of observation. This was accompanied by a fall in blood pressure of approximately 10 mm Hg in both sets of drug arms, with a slightly greater decrease in diastolic blood pressure with ASAQ compared with chloroquine, which had also resolved by day 7.

### Heart rate—Individual patient data meta-analysis

Heart rate data from ECG interval measurements were available from 2,680 individuals: 1,101 aged ≥12 years and 1,579 aged <12 years. Separate multivariable linear mixed effects models were fitted to heart rate data for these 2 age groups.

**Fig 5:**
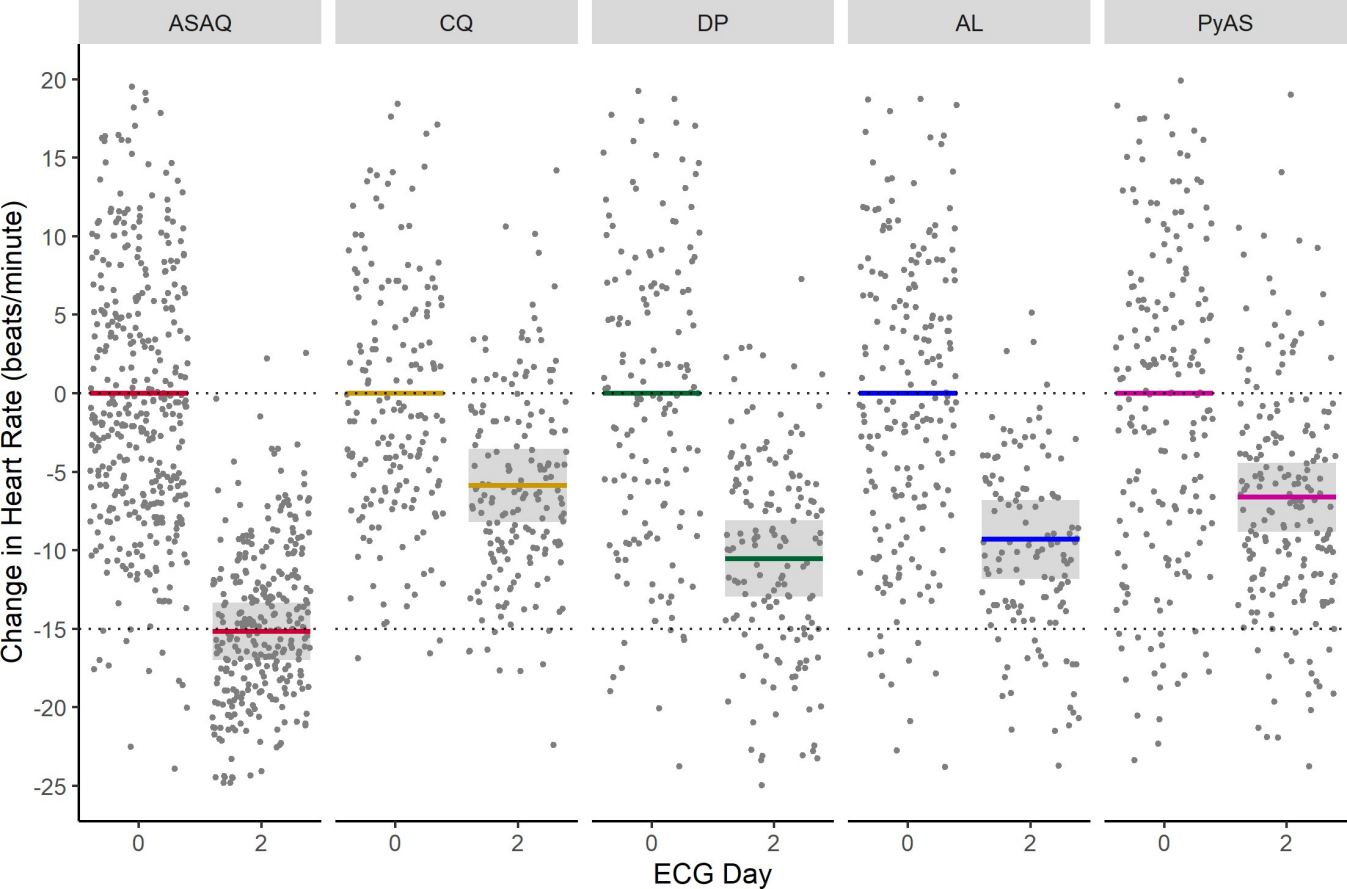
Change in heart rate after antimalarial treatment for uncomplicated malaria in adolescents and adults (age ≥12 years). Change in heart rate after treatment with the antimalarials artesunate-amodiaquine (ASAQ), chloroquine (CQ), dihydroartemisinin-piperaquine (DP), artemether-lumefantrine (AL), and pyronaridine-artesunate (PyAS) in adolescents and adults aged ≥12 years from a multivariable linear mixed effects regression model adjusting for sex, body temperature, and individual study/site/person effects. Lines indicate means with colour representing antimalarial treatment arm, shaded areas denote 95% confidence intervals, and dots are partial residuals.

Results from the model for adolescents and adults (≥12 years) are summarised in Table 4. ASAQ was associated with the greatest mean decrease in heart rate from baseline to day 2 (15.2 beats/minute, 95% CI: 13.4 to 17.0), followed by dihydroartemisinin–piperaquine (10.5 beats/minute, 95% CI: 7.7 to 13.3) and artemether–lumefantrine (9.3 beats/minute, 95% CI: 6.4 to 12.2), then pyronaridine–artesunate (6.6 beats/minute, 95% CI: 4.0 to 9.3) and chloroquine (5.9 beats/minute, 95% CI: 3.2 to 8.5) after adjustment for body temperature, and sex (Fig 5). The larger decrease in heart rate after ASAQ was both clinically and statistically significant versus the comparator antimalarials (artemether–lumefantrine: *p* < 0.001, chloroquine: *p* < 0.001, dihydroartemisinin–piperaquine: *p* = 0.0013, and pyronaridine–artesunate: *p* < 0.001). Body temperature was associated with a mean decrease in heart rate of 8.1 beats/minute (95% CI: 7.5 to 8.7) per 1°C of defervescence with all other variables held constant. Males had lower heart rates than females by a mean of 7.8 beats/minute (95% CI: 6.4 to 9.3). The differences in mean heart rates between ASAQ and comparator antimalarials at baseline were neither clinically nor statistically significant after adjustment for body temperature and sex.

**Table 4:** Multivariable linear mixed effects analysis of heart rate after antimalarial treatment in adolescents and adults (age ≥12 years).

Variable	Number of observations	Adjusted estimate (95% CI)	*p*-value
Change from baseline after ASAQ	689	−15.17 (−16.98 to −13.35) beats/minute	<0.001
Change from baseline versus ASAQ, by antimalarial treatment arm	2,041		
ASAQ	689	Reference	
CQ	345	9.30 (6.63 to 11.98) beats/minute	<0.001
DP	298	4.64 (1.82 to 7.45) beats/minute	0.0013
AL	328	5.86 (2.99 to 8.73) beats/minute	<0.001
PA	381	8.54 (5.94 to 11.13) beats/minute	<0.001
Baseline measurement versus ASAQ, by antimalarial treatment arm	1,096		
ASAQ	384	Reference	
CQ	172	−0.75 (−3.40 to 1.91) beats/minute	0.5816
DP	149	−0.07 (−3.12 to 2.97) beats/minute	0.9620
AL	200	−0.41 (−3.14 to 2.31) beats/minute	0.7672
PA	191	−2.23 (−5.20 to 0.74) beats/minute	0.1408
Body temperature, per 1°C increase	2,041	8.14 (7.54 to 8.73) beats/minute	<0.001
Sex	2,041		
Female	829	Reference	
Male	1,212	−7.84 (−9.27 to −6.41) beats/minute	<0.001

AL, artemether–lumefantrine; ASAQ, artesunate–amodiaquine; CQ, chloroquine; DP, dihydroartemisinin–piperaquine; PA, pyronaridine–artesunate.

**Fig 6:**
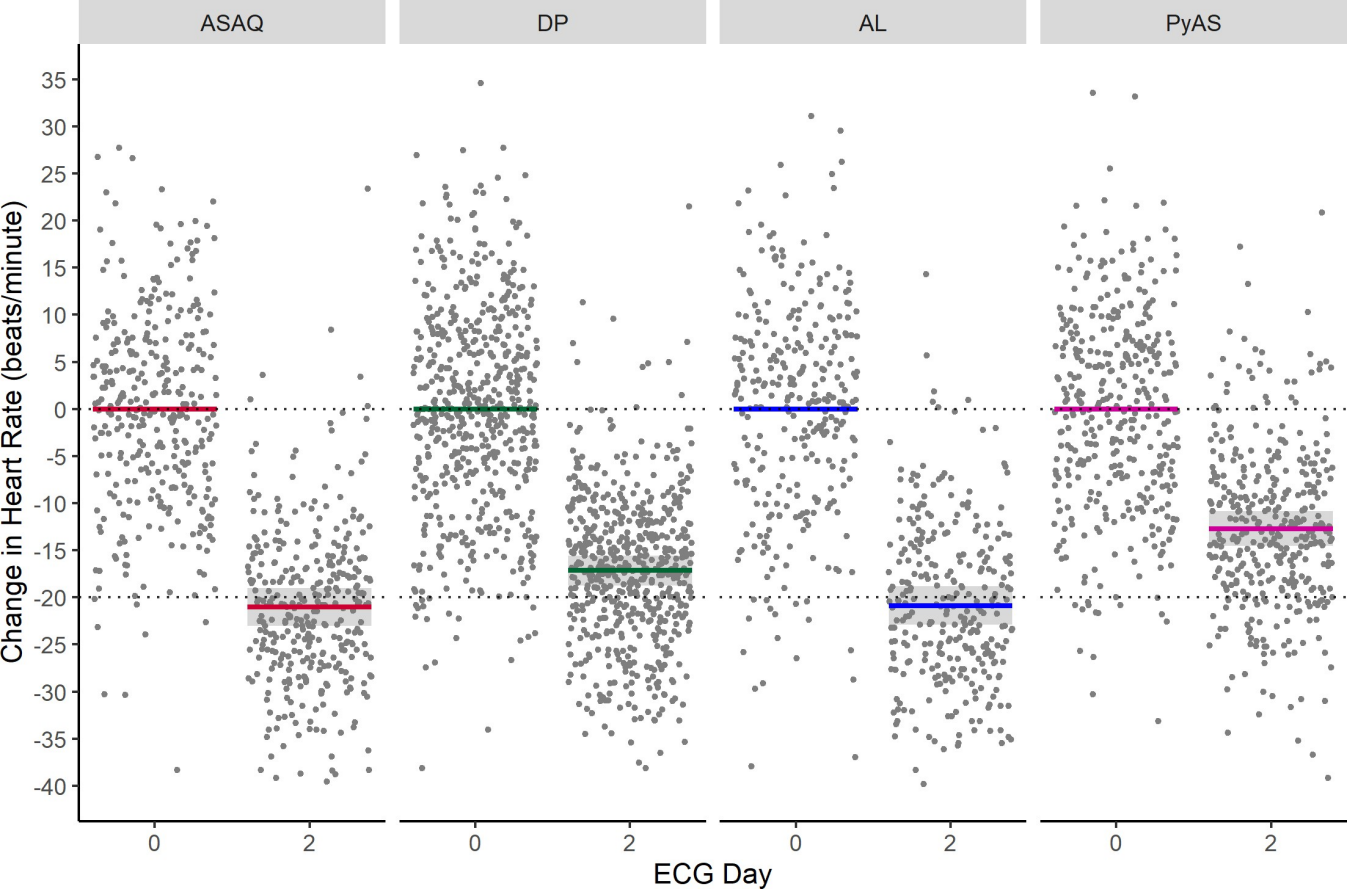
Change in heart rate after antimalarial treatment for uncomplicated malaria in children (age <12 years). Change in heart rate after treatment with the antimalarials artesunate-amodiaquine (ASAQ), dihydroartemisinin-piperaquine (DP), artemether-lumefantrine (AL), and pyronaridine-artesunate (PyAS) in children aged <12 years from a multivariable linear mixed effects regression model adjusting for age, body temperature, and individual study/site/person effects. Lines indicate means with colour representing antimalarial treatment arm, shaded areas denote 95% confidence intervals, and dots are partial residuals.

Results from the model for children (<12 years) are summarised in Table 5. ASAQ was again associated with the greatest mean decrease in heart rate from baseline to day 2 (21.0 beats/minute, 95% CI: 19.0 to 23.0), similar to that seen with artemether–lumefantrine (20.9 beats/minute, 95% CI: 18.3 to 23.5), followed by dihydroartemisinin–piperaquine (17.2 beats/minute, 95% CI: 14.9 to 19.4) then pyronaridine–artesunate (12.7 beats/minute, 95% CI: 10.2 to 15.1) (Fig 6). The decrease in heart rate seen after ASAQ was larger than after dihydroartemisinin–piperaquine (*p* < 0.001) and pyronaridine–artesunate (*p* < 0.001), but was neither clinically nor statistically significantly different from that observed after artemether–lumefantrine (*p* = 0.9015). Body temperature was associated with a mean decrease in heart rate of 5.5 beats/minute (95% CI: 4.9 to 6.1) per 1°C of defervescence and age with a mean decrease of 3.8 beats/minute (95% CI: 3.6 to 4.0) per year of childhood with all other variables held constant. The differences in heart rates between ASAQ and comparator antimalarials at baseline were neither clinically nor statistically significant after adjustment for body temperature and age.

**Table 5:** Multivariable linear mixed effects analysis of heart rate after antimalarial treatment in children (age <12 years).

Variable	Number of observations	Adjusted estimate (95% CI)	*p*-value
Change from baseline after ASAQ	674	−21.03 (−23.01 to −19.04) beats/minute	<0.001
Change from baseline versus ASAQ, by antimalarial treatment arm	3,151		
ASAQ	674	Reference	
DP	1,131	3.87 (1.63 to 6.12) beats/minute	<0.001
AL	596	0.16 (−2.43 to 2.76) beats/minute	0.9015
PA	750	8.33 (5.88 to 10.77) beats/minute	<0.001
Baseline measurement versus ASAQ, by antimalarial treatment arm	1,579		
ASAQ	338	Reference	
DP	567	−0.09 (−2.11 to 1.93) beats/minute	0.9318
AL	299	0.59 (−1.92 to 3.10) beats/minute	0.6433
PA	375	−0.75 (−2.98 to 1.49) beats/minute	0.5132
Body temperature, per 1°C increase	3,151	5.49 (4.91 to 6.07) beats/minute	<0.001
Age, per 1-year increase	3,151	−3.77 (−3.99 to −3.55) beats/minute	<0.001

AL, artemether–lumefantrine; ASAQ, artesunate–amodiaquine; DP, dihydroartemisinin–piperaquine; PA, pyronaridine–artesunate.

## Discussion

This large analysis of the cardiovascular effects of amodiaquine in the treatment of malaria provides a comprehensive assessment of its effects on heart rate and ventricular repolarisation. Data were available from 5,509 participants in trials with amodiaquine treatment arms including 2,681 individuals with ECG monitoring. These trials compared ASAQ to the most widely used front-line oral antimalarials for uncomplicated *P*. *falciparum* (artemether–lumefantrine and dihydroartemisinin–piperaquine) and *P*. *vivax* (chloroquine) malaria as well as the latest ACT indicated for use in treatment of both *P*. *falciparum* and *P*. *vivax* malaria (pyronaridine–artesunate).

### Corrected QT interval

ASAQ was found to prolong the rate corrected QT interval transiently regardless of correction factor used. This rate corrected QT interval prolongation after ASAQ was more than that after pyronaridine–artesunate and artemether–lumefantrine but less than after dihydroartemisinin–piperaquine and chloroquine including after adjustment for body temperature, sex, and age. However, these differences were small, of unclear clinical significance, and did not reach statistical significance when analysed at the level of the individual single-site trial [20].

The clinical significance of QT interval prolongation is as an imperfect surrogate marker for the risk of developing torsades de pointes (TdP), a polymorphic ventricular tachycardia that may degenerate into ventricular fibrillation and cause sudden cardiac death. In contrast to how it is often used, the relationship between QT prolongation and proarrhythmia is inconsistent and qualitative rather than quantitative: Drugs that prolong the QT interval range from having potent torsadogenic activity (e.g., halofantrine) to no proarrhythmic action and can even be anti-arrhythmic (e.g., amiodarone). Moreover, TdP can occur without QT prolongation and many QT prolonging drugs do not cause TdP [41]. These complexities are due in part to the challenges of quantifying multichannel blockade and the refractory periods of the cardiac action potential on the surface ECG. The lack of consensus about thresholds and definitions of drug-induced QT prolongation reflects these limitations. ICH-recommended categorical analyses using multiple thresholds (absolute value: >500, >480, and >450 milliseconds; change from baseline: >60 and >30 milliseconds) are proposed to be a reasonable approach to address this uncertainty [31] and provide useful information about the QT prolonging profile of a drug. However, individual QT values above these thresholds have been frequently misinterpreted as being clinical complications or reported as “serious adverse events” necessitating intervention even though only QT values of >500 milliseconds—corrected or uncorrected for heart rate—are considered definitely abnormal [31,42]. Similarly, the >20 and >5 millisecond thresholds for mean prolongations in ICH guidelines do not identify drugs as proarrhythmic. They indicate an increased or possibly increased likelihood, respectively, of a drug being proarrhythmic and the need for close attention in drug development to identify arrhythmic events in clinical trials [31]. All this information contributes towards risk–benefit evaluations, which ultimately determine whether the drug continues to be developed by the pharmaceutical company or receives regulatory approval and how it is used.

In the case of the quinoline and structurally related antimalarials, QT prolongation is a well-known feature of this drug class. Indeed, QT prolongation with TdP was once termed the “quinidine effect” [43], and, after initial use as an antimalarial, quinidine’s main use for the past 100 years has been as an anti-arrhythmic. As recommended in ICH guidance [31], comparisons within the drug class are valuable to define further any proarrhythmic risk. Moreover, evaluation of the cardiotoxicity of antimalarials is aided by decades of clinical experience with the widespread deployment of these drugs throughout the malaria endemic world and hundreds of millions of patients treated. Of the oral quinoline and quinoline-like antimalarials, only halofantrine is considered to have an unacceptable risk of arrhythmia when used for malaria treatment, and it has never been recommended for use by WHO [13]. This risk was first identified—after less extensive use than current front-line antimalarials—following a sudden death [44], which was soon followed by reports of further deaths as well as ventricular fibrillation, TdP, conduction disturbances, and extreme mean QT interval prolongation [45] (>60 milliseconds) [13].

All current front-line antimalarials, including ASAQ, result in mean corrected QT prolongations from baseline of approximately 20 milliseconds or less at standard malaria doses and when administered according to the manufacturer’s recommendations (e.g., taking dihydroartemisinin–piperaquine when fasted). The data on proarrhythmic risk of drugs with mean QT interval prolongation of 20 milliseconds or less are considered inconclusive, while those associated with prolongation of more than 20 milliseconds have a higher risk of clinically significant arrhythmias being captured during drug development [31]. In our analysis, chloroquine had the greatest mean corrected QT interval prolongation and was the only drug with a mean prolongation of just above 20 milliseconds. It is one of the most widely used drugs in humans, with hundreds of metric tons dispensed annually since the 1950s and a terminal elimination half-life of over a month. Chloroquine could well be the drug to which humans have been most exposed. It remains a first-line treatment for *P*. *vivax* malaria. While chloroquine has been associated very rarely with sudden deaths and TdP, this has been in overdose or for chronic indications other than treatment of malaria [13]. This analysis concentrated on the QT interval, which is the sum of the QRS and JT intervals, representing ventricular depolarisation and repolarisation, respectively. The quinoline antimalarials affect several ion channels. Chloroquine delays atrioventricular conduction and ventricular depolarisation in addition to repolarisation, although the delay in repolarisation has been of greatest concern [15].

Dihydroartemisinin–piperaquine had the next longest mean QT prolongation of just under 20 milliseconds in our analysis of malaria patients, and this was similar to the prolongation seen in healthy volunteers receiving single [14] or repeated [46] courses of the drug. Dihydroartemisinin–piperaquine has now been used extensively with millions of doses distributed for treatment and prevention of malaria, including as part of large mass drug administration programmes. The risk of sudden unexplained death after dihydroartemisinin–piperaquine is no higher than the baseline population risk [16], and, despite having undergone the most extensive cardiac safety evaluation of any antimalarial in history [16,18,47], dihydroartemisinin–piperaquine has never been definitively associated with ventricular fibrillation or TdP [13]. Artemether–lumefantrine was the first fixed-dose ACT to be prequalified by WHO and has also been studied intensively. As with dihydroartemisinin–piperaquine, the QT prolongation after artemether–lumefantrine in our analysis was small and similar to that observed in healthy volunteers [14]. Artemether–lumefantrine has not been associated with arrhythmic adverse events [13,18]. Further safety results from the large Phase IV study of pyronaridine–artesunate (NCT03201770) are awaited.

ASAQ is associated with an intermediate degree of QT prolongation between that after dihydroartemisinin–piperaquine and that after artemether–lumefantrine. Compared with other antimalarials, ASAQ induces greater bradycardia of clinical and statistical significance in adolescents and adults (≥12 years) but the bradycardia observed in children was not different to that with the other antimalarial drug treatments. While bradycardia is an important risk factor for TdP [42], amodiaquine has not been associated with sudden deaths or TdP [13,18] even with extensive use, particularly in SMC [48] (at least 85 million and 102.7 million treatments distributed in 2018 and 2019, respectively) in young children. Caution is advised when using amodiaquine in patients aged ≥12 years with serious conduction disorders and concomitant use of other drugs that slow the heart rate such as ivabradine, beta-blockers, and calcium channel blockers, particularly in the presence of other risk factors for TdP such as structural heart disease and other QT prolonging medications.

### Heart rate correction and nondrug factors affecting the QT interval

The QT interval shortens as heart rate increases. In our analysis, age group–specific corrections for each individual study provided the most effective heart rate adjustment for the QT interval. In line with what is known from healthy populations [31,49], the commonly used Bazett and Fridericia corrections both provided suboptimal heart rate correction in malaria with clinically significant residual heart rate dependency of the QT interval, although in opposite directions: the Bazett correction overestimated absolute QT interval values and underestimated QT interval changes, while the Fridericia correction underestimated absolute QT values and overestimated QT interval changes. The greater, possibly drug-related, decrease in heart rate seen after amodiaquine further augments the effects of any heart rate dependency on the corrected QT interval, confounding comparison of corrected QT interval changes between amodiaquine-containing antimalarials and those which are associated with smaller changes in heart rate, particularly in the treatment of malaria where disease recovery is itself associated with resolution of tachycardia.

Although an age group–specific correction for each study resulted in satisfactory heart rate correction of the QT interval in our analysis at the population level, it may be possible to optimise heart rate corrections further by deriving corrections specific to other factors that affect both the QT interval and heart rate, such as sex and severity of malaria illness in addition to age group [24]. In our analysis, the QT–RR relationship changed with day of treatment with the correction exponent being larger at peak disease and lower during recovery. We have also found previously that the correction exponent increases with malaria severity [24]. However, population-specific correction factors require specialist input to derive and are of unclear generalisability to individual patients at the point of care as the QT–RR relationship is highly individual [50]. Individual heart rate corrections are often not practicable in malaria trials or clinical practice because of resource limitations on ECG monitoring and are also complicated by confounding from disease recovery.

For comparison of QT values to assess the relative difference between treatment arms in randomised controlled trials where factors known to affect the QT interval (age, sex, body temperature changes, and malaria recovery) are balanced between arms, the use of the Bazett and Fridericia corrections does provide interpretable information. However, if absolute values of corrected QT interval prolongation are of interest, then optimising heart rate correction with appropriate adjustment for the other factors which affect the QT interval would be necessary to obtain the true drug-related effect.

### Heart rate

In adolescents and adults (≥12 years), ASAQ was associated with a higher incidence of sinus bradycardia and a greater mean reduction in heart rate of clinical and statistical significance compared with front-line antimalarials, even after adjusting for body temperature and sex. More than 50% of adults developed sinus bradycardia, 15% of these to a degree which could be symptomatic. This heart rate reduction appeared to be concentration dependent, reaching its nadir around the time of maximum amodiaquine and desethylamodiaquine concentrations before returning to baseline levels 3 to 5 days later. In children (<12 years), ASAQ was again associated with the greatest mean heart rate reduction of the antimalarials under investigation, although, unlike in adolescents and adults, this was similar to that observed after artemether–lumefantrine, and it did not result in a clinically significant increase in the incidence of sinus bradycardia versus comparator antimalarials. These decreases in heart rate were accompanied by a small and likely concentration-dependent drop in blood pressure, similar to that observed following chloroquine, which also returned to baseline levels over the same timescale. This overriding of baroreceptor reflex activity—where blood pressure reduction usually decreases baroreflex activation to cause an increase in heart rate—provides further evidence of a pharmacological effect in addition to malaria recovery-related changes.

In keeping with our findings, 16 (64%) of 20 patients had sinus bradycardia after amodiaquine monotherapy at total doses of 30 to 35 mg/kg over 3 days in the only cardiac safety study of amodiaquine in adults with uncomplicated malaria [19], which was conducted in Cameroon. In the few studies [19,20] of amodiaquine with electrocardiographic monitoring not included in this meta-analysis and in case reports [51,52], sinus bradycardia after amodiaquine has been recorded almost exclusively (24/25) in patients ≥12 years around the time of expected maximum drug concentration of amodiaquine and desethylamodiaquine. This bradycardia can be symptomatic with easy fatiguability [51] and dizziness when accompanied by hypotension [52]. Higher day 3 desethylamodiaquine concentrations have previously been associated with higher rates of bradycardia [20], while higher day 7 concentrations have been less consistently associated with fatigue [53] and vomiting [53].

Drug-related bradycardia and concomitant hypotension may contribute to the higher incidence of asthenia and asthenia-like reactions after amodiaquine compared with other antimalarials. The symptoms of bradycardia and hypotension include dizziness, weakness, and fatiguability. Amodiaquine-associated asthenia has been observed more frequently in adolescents and adults ≥12 years than in children [54] and is typically self-limiting, lasting for about 3 days after the end of amodiaquine treatment [54]. Pregnant women with malaria appear to be more susceptible [52,55], possibly because of gestational-related changes in heart rate and blood pressure [56]. As asthenia is also a common feature of clinical malaria, discriminating between adverse events that are symptoms of the acute illness and treatment-emergent signs and symptoms, such as through active elicitation of carefully defined adverse events of special interest at prespecified time points, would be especially important to improve detection of this adverse drug reaction [57].

Possible molecular mechanisms are both neurological and cardiovascular. Amodiaquine exhibits potent reversible acetylcholinesterase inhibition [58] (more than chloroquine and much more than mefloquine) [59,60], which could potentiate the effects of parasympathetic activation and account for vagally mediated bradycardia and dizziness as well as vomiting and diarrhea. Like hydroxychloroquine [61], amodiaquine also reduces the pacemaker “funny” current at the sinoatrial node, which directly causes bradycardia.

Further clinical characterisation of the possible relationship between asthenia and bradycardia through systematic cardiovascular vital sign monitoring and exercise testing after amodiaquine would help us better understand the contribution of the cardiovascular system to this common side effect of amodiaquine. Prospective validation of these cardiovascular findings in adult healthy volunteers (NCT04080895) is ongoing.

### Limitations

Our study is limited by its retrospective design, measurement of subintervals of the QT interval in only three of the four included studies, the unavailability of individual patient-level data on adverse events for the majority of included participants, and heterogeneity in adverse event data collection methodology of the two studies for which individual patient-level adverse event data were shared. However, no serious cardiovascular events were documented in any of the studies of amodiaquine from which individual patient data were sought. Data on the other ECG intervals following amodiaquine for malaria and results of ongoing prospective safety studies will be reported elsewhere.

## Conclusions

Amodiaquine causes the greatest reduction of heart rate of the front-line antimalarials, resulting in sinus bradycardia more frequently in adolescents and adults than in children. It also prolongs the QT interval less than chloroquine and piperaquine but more than lumefantrine and pyronaridine. Despite these effects, there have been no sudden deaths or clinically significant arrhythmias reported throughout its extensive use in the treatment and prevention of malaria [13,18,48]. Our analysis supports the cardiovascular safety of amodiaquine and structurally related antimalarials at WHO-recommended dose regimens alone or in ACTs for malaria-related indications. At the same time, amodiaquine is associated with a higher incidence of asthenia and dizziness compared with other antimalarials, particularly in adults, to which its bradycardic effects may contribute. Further clinical and molecular definition of the effects of amodiaquine on the cardiovascular system are warranted to develop measures to improve the tolerability of this important antimalarial.

## Supporting information

S1 Checklist PRISMA-IPD Checklist. PRISMA-IPD, Preferred Reporting Items for Systematic Reviews and Meta-Analyses of Individual Patient Data.
(DOCX)

S1 Appendix Supplementary Methods and Results. (DOCX)

## Abbreviations

ACT: artemisinin-based combination therapy
ASAQ: artesunate–amodiaquine
bpm: beats per minute
COVID-19: Coronavirus Disease 2019
ICH: International Council for Harmonisation of Technical Requirements for Pharmaceuticals for Human Use
PRISMA-IPD: Preferred Reporting Items for Systematic Reviews and Meta-Analyses of Individual Patient Data
PROTECT: Pharmacoepidemiological Research on Outcomes of Therapeutics by a European Consortium
SMC: seasonal malaria chemoprevention
TdP: torsades de pointes
WANECAM: West African Network for Clinical Trials of Antimalarial Drugs
WHO: World Health Organization
