# Supplementary material for: The cardiovascular effects of amodiaquine and structurally related antimalarials: An individual patient data meta-analysis
Source: PLoS Med. 2021 Sep 7;18(9):e1003766. doi: 10.1371/journal.pmed.1003766 (PMC8454971; doi:10.1371/journal.pmed.1003766)
Supplement: S1 Appendix — (DOCX) [file pmed.1003766.s002.docx]

**Supplementary Appendix**

**The Cardiovascular Effects of Amodiaquine and Structurally Related Antimalarials:
An Individual Patient Data Meta-analysis**

Xin Hui S Chan, Ilsa L Haeusler, Yan Naung Win, James Pike, Borimas Hanboonkunupakarn,
Maryam Hanafiah, Sue J Lee, Abdoulaye Djimde, Caterina I Fanello, Jean-René Kiechel,

Marcus VG Lacerda, Bernhards Ogutu, Marie A Onyamboko, André M Siqueira,

Elizabeth A Ashley, Walter RJ Taylor, Nicholas J White

**Table of Contents**

[Supplementary Methods 4](#_Toc79807141)

[Study-Level Data Extraction 4](#_Toc79807142)

[Individual Patient-Level Data Processing 4](#_Toc79807143)

[Individual Patient-Level Data Standardisation 4](#_Toc79807144)

[ECG Intervals 4](#_Toc79807145)

[RR Interval & Heart Rate 4](#_Toc79807146)

[QT/QTc Interval 4](#_Toc79807147)

[QRS Interval 5](#_Toc79807148)

[PR Interval 5](#_Toc79807149)

[Demographics 5](#_Toc79807150)

[Age 5](#_Toc79807151)

[Weight 5](#_Toc79807152)

[Vital Signs 5](#_Toc79807153)

[Pulse Rate 5](#_Toc79807154)

[Blood Pressure 5](#_Toc79807155)

[Temperature 5](#_Toc79807156)

[Laboratory Parameters 5](#_Toc79807157)

[Parasitaemia 5](#_Toc79807158)

[Haemoglobin 6](#_Toc79807159)

[Antimalarial Drug-Related Parameters 6](#_Toc79807160)

[Milligram per Kilogram Dose 6](#_Toc79807161)

[Vomiting & Repeated Doses 6](#_Toc79807162)

[Concomitant Medications 6](#_Toc79807163)

[Antimalarial Pre-treatment 6](#_Toc79807164)

[Individual Patient-Level Data Integrity Checks 6](#_Toc79807165)

[Data Analysis 7](#_Toc79807166)

[Variable Selection 8](#_Toc79807167)

[Model Formulation 8](#_Toc79807168)

[Meta-analysis – Corrected QT Interval Model 8](#_Toc79807169)

[Meta-analysis – Heart Rate Models 8](#_Toc79807170)

[Supplementary Results 12](#_Toc79807171)

[Study Characteristics 12](#_Toc79807172)

[Population Characteristics 16](#_Toc79807173)

[Corrected QT Interval Analyses 19](#_Toc79807174)

[Heart Rate Analyses 23](#_Toc79807175)

[Cardiovascular Vital Signs Analyses 25](#_Toc79807176)

[References 27](#_Toc79807177)

**List of Figures**

[Figure A: Directed Acyclic Graph of Factors Affecting the Electrocardiographic QT Interval in Malaria after Amodiaquine Treatment 9](#_Toc80532688)

[Figure B: Directed Acyclic Graph of Factors Affecting the Heart Rate in Malaria in Adults after Amodiaquine Treatment 10](#_Toc80532689)

[Figure C: Directed Acyclic Graph of Factors Affecting the Heart Rate in Malaria in Children after Amodiaquine Treatment 11](#_Toc80532690)

[Figure D: Histogram of Total Amodiaquine Dose Received 16](#_Toc80532691)

[Figure E: Total Amodiaquine Dose Received by Individual Body Weight 17](#_Toc80532692)

[Figure F: Total Non-Amodiaquine Quinoline and Structurally Related Antimalarial Dose Received by Individual Body Weight 18](#_Toc80532693)

[Figure G: Corrected QT Interval and RR Interval Relationships by Correction Method, Treatment Day, and Individual Study 19](#_Toc80532694)

[Figure H: Pulse Rate and Blood Pressure Measurements after Artesunate-Amodiaquine and Chloroquine for *P. vivax* Malaria in Brazilian Adults (≥12 years) and Children (<12 years) 25](#_Toc80532695)

[Figure I: Pulse Rate and Blood Pressure Measurements after Artesunate-Amodiaquine for *P. falciparum* Malaria in Kenyan Adults (≥18 Years) 26](#_Toc80532696)

**List of Tables**

[Table A: Study-Specific Correction Exponents by Age Group 7](#_Toc80532784)

[Table B: Bradycardia Thresholds by Age Group 7](#_Toc80532785)

[Table C: Additional Characteristics of Included Studies 12](#_Toc80532786)

[Table D: Weight-based Drug Dosing Tables of Included Studies 13](#_Toc80532787)

[Table E: Comparison of Included Studies and Excluded Studies 14](#_Toc80532788)

[Table F: Risk of Bias Assessment of Included and Excluded Studies 15](#_Toc80532789)

[Table G: Summary Statistics of Corrected QT Interval Measurements from ECG Interval Analysis by Drug Arm and Time Point 20](#_Toc80532790)

[Table H: Summary Statistics of Heart Rate Measurements from ECG Interval Analyses of Individual Studies by Drug Arm and Time Point 23](#_Toc80532791)

# Supplementary Methods

## Study-Level Data Extraction

The following information was extracted from study publications, reports, and protocols, and where necessary, requested from study investigators:

1. Study characteristics: study design, year of publication, recruitment period, location, participant population, antimalarial treatment indication, number of days of follow-up, participant inclusion and exclusion criteria, number of participants who had ECG monitoring
2. Antimalarial dosing regimen: route of administration, course length, dosing frequency, drug formulation, drug manufacturer, dosing table, level of supervision, food intake around time of dosing
3. Antimalarial drug concentration measurement: drug measurement timepoints, units of measurement, biological matrix (capillary whole blood or venous plasma), assay type, assay limit of quantification
4. ECG measurement methodology: ECG measurement timepoints, centralised or study site-based, manual or automated, cardiologist or other physician reader, intermittent or continuous, other relevant details
5. Cardiovascular adverse events: sudden cardiac death, life-threatening ventricular tachyarrhythmias (ventricular fibrillation, ventricular tachycardia, torsade de pointes), other clinically significant arrhythmias or cardiovascular adverse events

## Individual Patient-Level Data Processing

Manual data entry was undertaken for datasets available only in printed format. Once digitised, individual patient-level datasets were converted into a standard file format using Stat/Transfer^1^ version 13.3.

## Individual Patient-Level Data Standardisation

This was implemented via a bespoke Application Programming Interface in Python version 3.7.2.

### ECG Intervals

Where the same ECG recording was measured by more than one set of readers, the measurements from the more specialist set^2^ of ECG readers were selected. Measurements from triplicate ECG recordings were averaged. Only measurements from intermittent ECG readings were used.

#### RR Interval & Heart Rate

RR intervals were standardised to units of milliseconds and transformed into heart rate based on the following formula as necessary:

- Heart rate = 60000/RR interval

#### QT/QTc Interval

Where only corrected QT intervals were available, uncorrected QT intervals were calculated as follows:

- $QT = QTcB*\sqrt{RR}$ as $QTcB =\frac{QT}{\sqrt{RR}}$ (Bazett’s correction formula)
- $QT = QTcF*\sqrt[3]{RR}$ as $QTcF =\frac{QT}{\sqrt[3]{RR}}$ (Fridericia’s correction formula)

where RR intervals are in units of seconds

#### QRS Interval

QRS interval measurements were extracted as standardised to units of milliseconds, where available.

#### PR Interval

PR interval measurements were extracted as standardised to units of milliseconds, where available.

### Demographics

#### Age

Age was extracted as standardised to units of years, and otherwise calculated based on the number of years between the subject’s date of birth and the date of the start of the study.

#### Weight

Individual body weight was extracted as standardised to units of kilograms.

### Vital Signs

#### Pulse Rate

Peripheral pulse rate, i.e. heart rate as measured from the peripheral pulse rather than the RR interval on the ECG, was extracted as standardised to units of beats per minute.

#### Blood Pressure

Supine systolic and diastolic blood pressure measurements were extracted as standardised to units of mmHg.

Erect systolic and diastolic blood pressure measurements were also extracted as standardised to units of mmHg, where these were available.

#### Temperature

Oral and tympanic body temperatures were extracted as documented in the original data^3^, and converted to units of degrees Celsius as required. Axillary body temperatures were extracted, converted to units of degrees Celsius as required, then standardised by the addition of 0.5°C to original readings.

Body temperature was standardised to units of degrees Celsius using the following formula:

- Temperature (°C) = [Temperature (°F) – 32] / 1.8

### Laboratory Parameters

#### Parasitaemia

The highest parasite density available for each timepoint was extracted.

Malaria parasite count measurements were standardised as parasite density per microlitre of blood according to the following formulae before being logarithmically transformed:

- Parasitaemia = (parasite count per 500 WBC / 500) * WBC count [if WBC count available]
- Parasitaemia = (parasite count per 500 WBC / 500) * 8000 [if WBC count missing]

where WBC counts are in units of mm^3^ of blood

- Parasitaemia = parasite count per 1000 RBC * 125.6 * haematocrit [if haematocrit available]
- Parasitaemia = parasite count per 1000 RBC * 125.6 * 33 [if haematocrit missing]

where haematocrit is in units of %

#### Haemoglobin

For studies in which only haematocrit was measured, haemoglobin was calculated as follows:

- Haemoglobin (g/dl) = [haematocrit (%) – 5.62] / 2.6 as
  Haematocrit (%) = 5.62 + 2.60 * haemoglobin (g/dl)^4^

### Antimalarial Drug-Related Parameters

#### Milligram per Kilogram Dose

The total milligram per kilogram (mg/kg) dose of quinoline or structurally related antimalarial received by each individual was extracted where available but was otherwise derived from available drug dosing data and study-specific weight-based dosing charts as follows:

- mg/kg dose = total dose received (mg) / weight (kg)
- mg/kg dose = total number of doses * number of tablets per dose * dose per tablet (mg) / weight (kg)

#### Vomiting & Repeated Doses

Vomiting after dosing and whether the treatment dose was repeated after vomiting were extracted as ‘present’ or ‘absent’. Where a dose was repeated, the date and time of the repeated dose were used to calculate time from dosing.

#### Concomitant Medications

Concomitant medications were extracted as a list of drug names as recorded in the original data if available.

#### Antimalarial Pre-treatment

Antimalarial pre-treatment was extracted as ‘present’ or ‘absent’ with the name of the pre-treatment drug extracted as free text into a separate column where available.

## Individual Patient-Level Data Integrity Checks

Individual patient data were checked for completeness, as well as for invalid, out-of-range, or inconsistent entries. Values incompatible with what would be observed in malaria clinical trials were considered missing. Queries were raised with study investigators and resolved where possible.

## Data Analysis

Table A: Study-Specific Correction Exponents by Age Group

|  | **Study Reference** | | | |
| --- | --- | --- | --- | --- |
| **Age Group (years)** | **Ndiaye 2011^5^** | **Ogutu 2014^6^** | **Siqueira 2017^7^** | **WANECAM 2018^8^** |
| 0.5 to <5 | N/A | N/A | N/A | 0.46 |
| 5 to <10 | N/A | N/A | N/A | 0.43 |
| 10 to <15 | 0.455 | N/A | 0.42 | 0.43 |
| ≥15 | 0.435 | 0.42 | 0.35 | 0.395 |

The correction exponent $\beta_{age}$ is the coefficient of $agegroup*logRR$ from the log-log linear regression:
$logQT \sim agegroup*logRR + sex + temperature +ECGday*drug+ (1│patient)$

In addition to antimalarial drug, the malaria disease and demographic variables included are those previously identified to have independent effects on the QT interval in malaria

Table B: Bradycardia Thresholds by Age Group

|  | **Heart Rate (beats/minute)** | |
| --- | --- | --- |
| **Age Group (years)** | **Lower Limit of Normal (10^th^ Percentile)** | **Lower Limit (5^th^ Percentile)** |
| 0.5 to 2 | 100 | 90 |
| 2 to <5 | 80 | 70 |
| 5 to <8 | 75 | 65 |
| 8 to <12 | 70 | 60 |
| 12 to <15 | 65 | 55 |
| ≥15 | 60 | 50 |

Adapted from systematic review of normal range of heart rate in 143,346 healthy children between birth and 18 years of age from 69 observational studies^9^

### Variable Selection

Variable selection was based on directed acyclic graphs (Figure A-C) of proposed causal relationships among collected variables informed by literature review and expert consultation used to determine minimal sufficient adjustment sets for regression modelling.

### Model Formulation

#### Meta-analysis – Corrected QT Interval Model

QTcS ~ drug*ECGday + temperature + agegroup*sex + (1│study/site/patient)

where $QTcS=\frac{QT}{{RR}^{\beta_{age}}}$ and RR is in units of seconds

#### Meta-analysis – Heart Rate Models

HR_adult ~ drug*ECGday + temperature + sex + (1│study/site/patient) [age ≥12 years]

HR_child ~ drug*ECGday + temperature + age + (1│study/site/patient) [age <12 years]

Figure A: Directed Acyclic Graph of Factors Affecting the Electrocardiographic QT Interval in Malaria after Amodiaquine Treatment

**
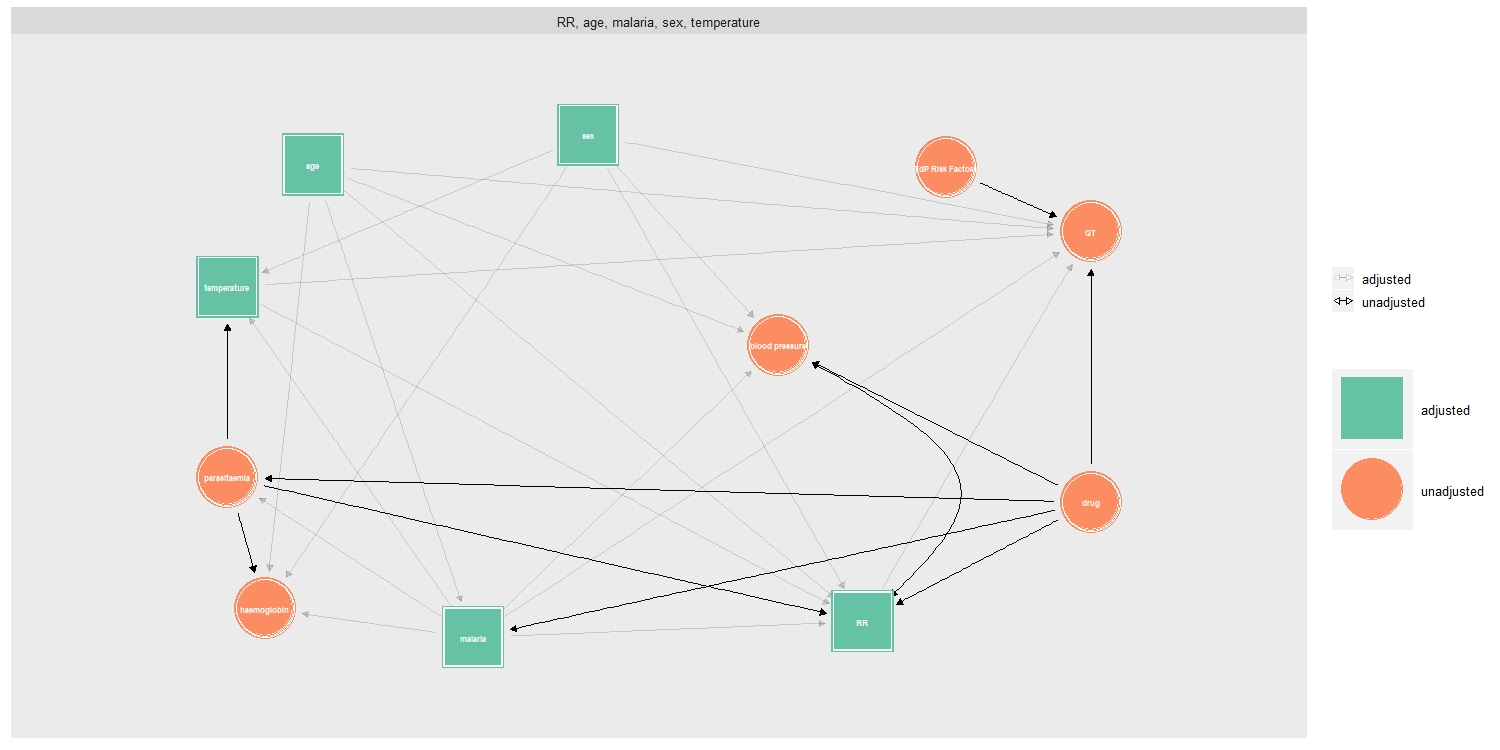
**

Directed acyclic graph generated in DAGitty^10^ describing proposed causal relationships among factors affecting the electrocardiographic QT interval in malaria after antimalarial treatment with amodiaquine showing minimal sufficient covariate adjustment set (facet label & green squares). Bidirectional arrows do not represent reciprocal causation but depict unobserved confounders. The minimal adjustment set consisting of malaria disease variables of malaria and temperature along with demographic covariates of age and sex were included as fixed effects in multivariable linear mixed effects analyses. A study-specific heart rate correction factor was used for RR interval-related confounding.

Figure B: Directed Acyclic Graph of Factors Affecting the Heart Rate in Malaria in Adults after Amodiaquine Treatment


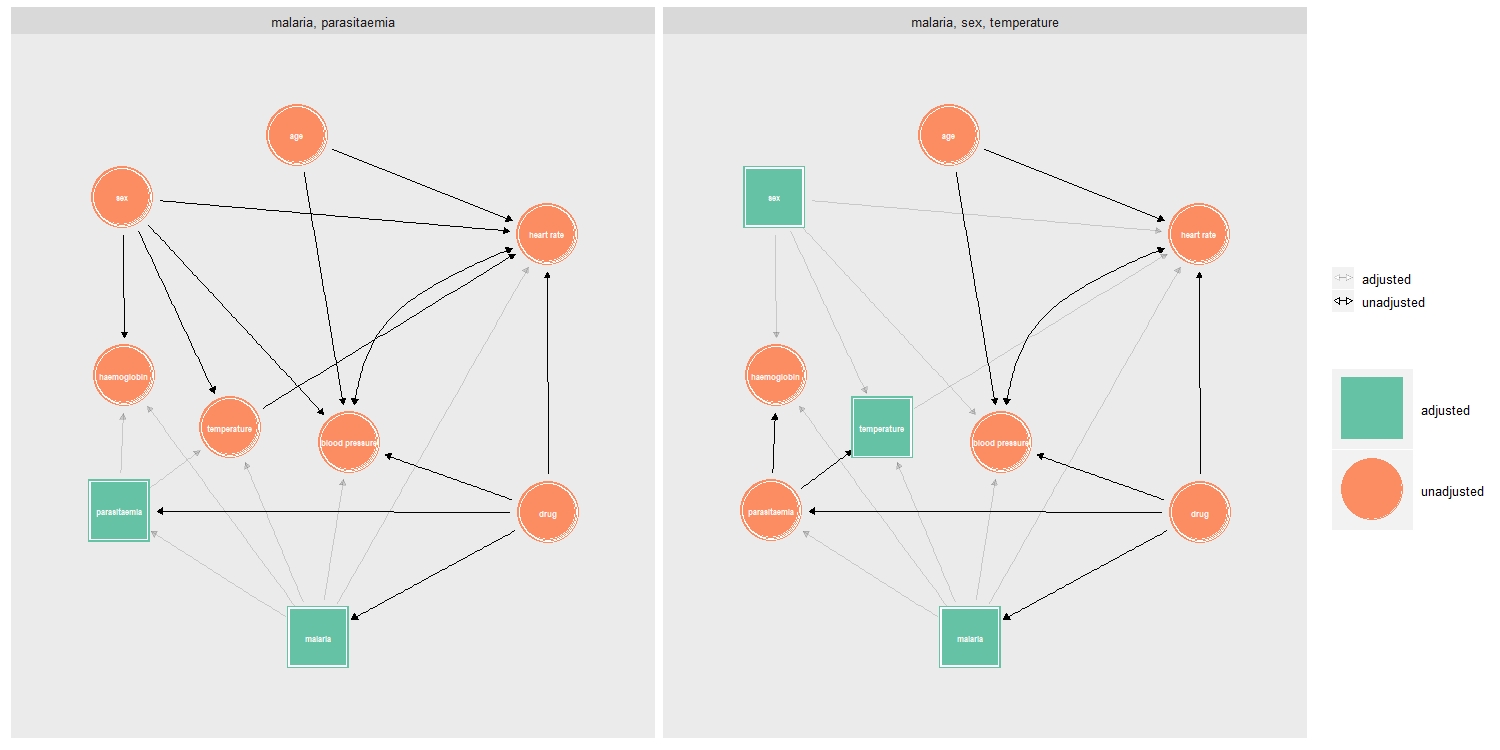


Directed acyclic graph generated in DAGitty^10^ describing proposed causal relationships among factors affecting the heart rate in malaria in adults after antimalarial treatment with amodiaquine showing minimal sufficient covariate adjustment set (facet label & green squares). Bidirectional arrows do not represent reciprocal causation but depict unobserved confounders. The minimal adjustment set consisting of disease variables of malaria and temperature along with the demographic covariate of sex were included as fixed effects in multivariable linear mixed effects analyses.

Figure C: Directed Acyclic Graph of Factors Affecting the Heart Rate in Malaria in Children after Amodiaquine Treatment


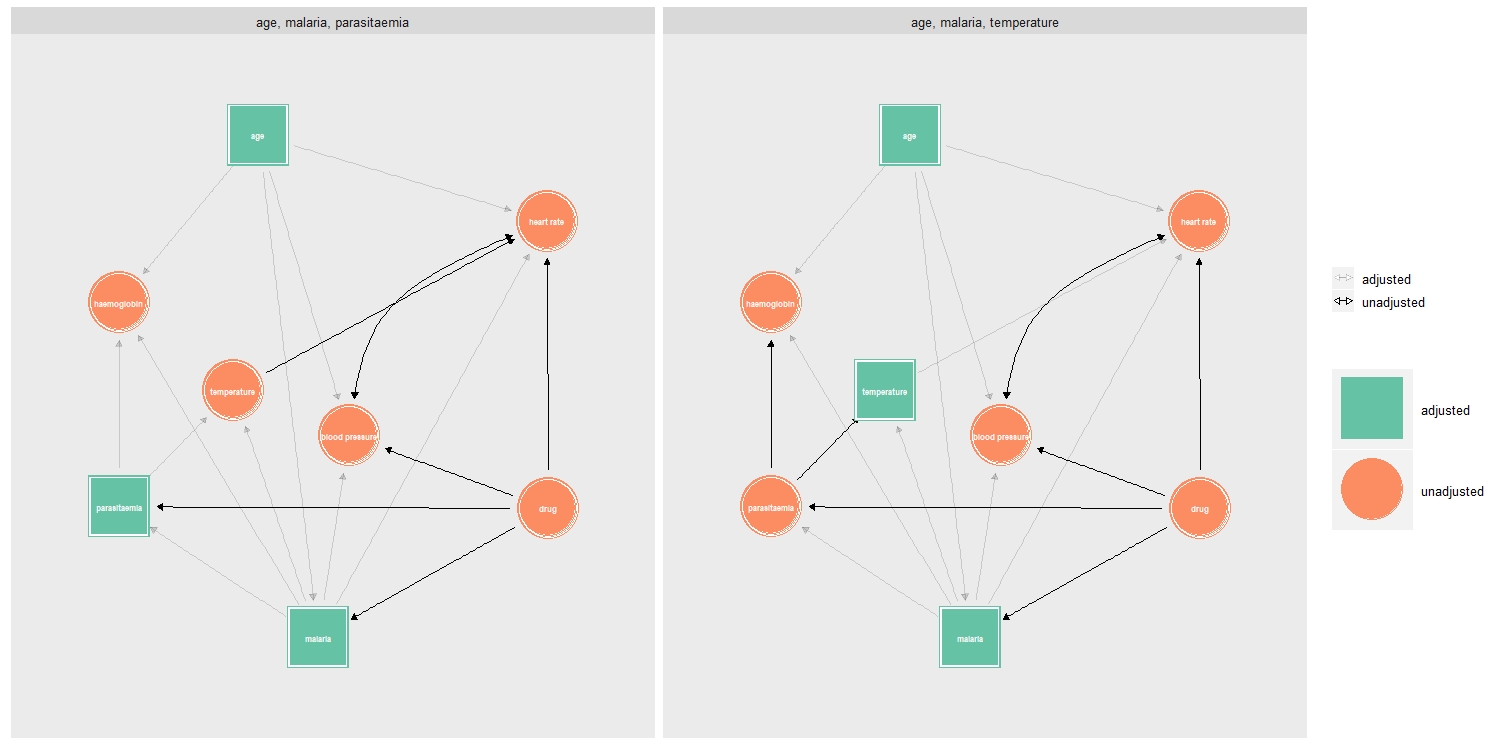


Directed acyclic graph generated in DAGitty^10^ describing proposed causal relationships among factors affecting the heart rate in malaria in children after antimalarial treatment with amodiaquine showing minimal sufficient covariate adjustment set (facet label & green squares). Bidirectional arrows do not represent reciprocal causation but depict unobserved confounders. The minimal adjustment set consisting of disease variables of malaria and temperature along with the demographic covariate of age were included as fixed effects in multivariable linear mixed effects analyses.

#

# Supplementary Results

## Study Characteristics

Table C: Additional Characteristics of Included Studies

| **Study Reference** | **Ndiaye 2011^5^** | **Ogutu 2014^6^** | **Siqueira 2017^7^** | **WANECAM 2018^8^** |
| --- | --- | --- | --- | --- |
| **Region** | Africa | Africa | Americas | Africa |
| **Recruitment** | 2007-2008 | 2007-2008 | 2011-2013 | 2011-2013 |
| **Adverse Event IPD** | Not available | Available | Available | Not available |
| **Antimalarial Pre-Treatment, Subjects  (% Total Subjects)** | Not available | 7 (13%) | 55 (14%) | Not available |
| **Antimalarial**  **Pre-Treatment Drug**  **(% Pre-treated Subjects)** | Not available | Chloroquine (71%)  or  Artemether-lumefantrine (29%) | Artesunate-mefloquine (95%)  or  Chloroquine (4%)  or  Artemether-lumefantrine (1%) +/-  Primaquine (96%) | Not available |
| **Cardiovascular Concomitant Medications, Subjects  (% Total Subjects)** | Not available | None | 18 (4.7%):  3 with more than one drug | Not available |
| **Cardiovascular Concomitant Medication  (% Concomitantly Treated Subjects)** | Not available | None | ACE inhibitor (39%)  Beta blocker (33%)  Thiazide or thiazide-like diuretic (22%)  A2R blocker (17%)  Calcium channel blocker (5.6%) | Not available |
| **ECG Measurement Location** | Centralised | Centralised | Site-based | Centralised |
| **ECG Measurement Reader** | Cardiologist | Cardiologist | Cardiologist | Cardiologist |
| **ECG Measurement Method** | Intermittent | Intermittent | Intermittent | Intermittent |
| **Temperature Measurement Method** | Axillary | Axillary | Axillary | Axillary or Oral |

IPD = individual participant-level data; ECG = electrocardiogram; ACE = angiotensin-converting enzyme; A2R = angiotensin II receptor

Table D: Weight-based Drug Dosing Tables of Included Studies

| Drug | Weight Band (kg) | Dose per day (mg) | Dose per 3-day course (mg) | Studies |
| --- | --- | --- | --- | --- |
| Artesunate-Amodiaquine FDC | 5 to <9 | 25:67.5 | 75:202.5 | Ndiaye 2011^5^, Ogutu 2014^6^,  Siqueira 2017^11^, WANECAM 2018^8,12^ |
|  | 9 to <18 | 50:135 | 150:405 |  |
|  | 18 to <36 | 100:270 | 300:810 |  |
|  | ≥36 | 200:540 | 600:1620 |  |
|  |  |  |  |  |
| Artesunate + Amodiaquine Non-FDC | ≥36 | 200 + 612 | 600 + 1836 | Ogutu 2014^6^ |
|  |  |  |  |  |
| Artemether-Lumefantrine FDC | 5 to <15 | 20:120 | 60:360 | Ndiaye 2011^5^, WANECAM 2018^8,12^ |
|  | 15 to <25 | 40:240 | 120:720 |  |
|  | 25 to <35 | 60:360 | 180:1080 |  |
|  | ≥35 | 80:480 | 240:1440 |  |
|  |  |  |  |  |
| Chloroquine | 5 to <10 | Day 1: 75 | 150 | Siqueira 2017^11^ |
|  |  | Days 2 & 3: 37.5 |  |  |
|  | 10 to <15 | Day 1: 150 | 300 |  |
|  |  | Days 2 & 3: 75 |  |  |
|  | 15 to <25 | Day 1: 150 | 450 |  |
|  |  | Days 2 & 3: 150 |  |  |
|  | 25 to <35 | Day 1: 300 | 900 |  |
|  |  | Days 2 & 3: 300 |  |  |
|  | 35 to <50 | Day 1: 450 | 1050 |  |
|  |  | Days 2 & 3: 300 |  |  |
|  | ≥50 | Day 1: 600 | 1500 |  |
|  |  | Days 2 & 3: 450 |  |  |
|  |  |  |  |  |
| Dihydroartemisinin-Piperaquine FDC | 5 to <7 | 10:80 | 30:240 | WANECAM 2018^8,12^ |
|  | 7 to <13 | 20:160 | 60:480 |  |
|  | 13 to <24 | 40:320 | 120:960 |  |
|  | 24 to <36 | 80:640 | 240:1920 |  |
|  | 36 to <75 | 120:960 | 360:2880 |  |
|  | ≥75 | 160:1280 | 480:3840 |  |
|  |  |  |  |  |
| Pyronaridine-Artesunate FDC | 5 to <8 | 60:20 | 180:60 | WANECAM 2018^8,12^ |
|  | 8 to <15 | 120:40 | 360:120 |  |
|  | 15 to <24 | 180:60 | 540:180 |  |
|  | 24 to <45 | 360:120 | 1080:360 |  |
|  | 45 to <65 | 540:180 | 1620:540 |  |
|  | >65 | 720:240 | 2160:720 |  |

FDC = fixed-dose combination

Table E: Comparison of Included Studies and Excluded Studies

|  | **Included Studies**  **(n = 4)** | **Excluded Studies**  **(n = 4)** |
| --- | --- | --- |
| **Antimalarial Treatment Indication, studies (%)** |  |  |
| Uncomplicated malaria | 4 (100%) | 4 (100%) |
| *P. falciparum* mono- or mixed infection | 3 (75%) | 4 (100%) |
| *P. vivax* mono-infection | 1 (25%) | 0 |
|  |  |  |
| **Amodiaquine Formulation, amodiaquine-containing drug arms (%)** |  |  |
| Fixed-dose combination therapy with artesunate | 4 (80%) | 0 |
| Non-fixed dose combination therapy | 1 (20%) | 4 (66.7%) |
| With artesunate | 0 | 3 (50%) |
| With sulfadoxine-pyrimethamine | 0 | 1 (16.7%) |
| Monotherapy | 0 | 2 (33.3%) |
|  |  |  |
| **Patient Population, studies (%)** |  |  |
| Adults & children | 3 (75%) | 0 |
| Adults only (male and female) | 1 (25%) | 1 (25%) |
| Adults only (males only) | 0 | 1 (25%) |
| Children only | 0 | 1 (25%) |
| Pregnant women only | 0 | 1 (25%) |
|  |  |  |
| **Geographical Region, studies (%)** |  |  |
| Africa | 3 (75%) | 4 (100%) |
| Americas | 1 (25%) | 0 |
|  |  |  |
| **Year Enrolment Completed, studies (%)** |  |  |
| 2007-2017 | 4 (100%) | 1 (25%) |
| Pre-2007 | 0 | 3 (75%) |
|  |  |  |
| **Study Design, studies (%)** |  |  |
| Randomised Controlled Trial | 4 (100%) | 3 (75%) |
| Cohort | 0 | 1 (25%) |
|  |  |  |
| **ECG Reader, studies (%)** |  |  |
| Cardiologist | 4 (100%) | 1 (25%) |
| Physician or other trained personnel | 0 | 1 (25%) |
| Machine | 0 | 1 (25%) |
| Not detailed | 0 | 1 (25%) |
|  |  |  |
| **Torsade de Pointes Risk Factors Excluded, studies (%)** | 3 (75%) | 2 (50%) |
|  |  |  |
| **Mean Age in Years, median (IQR)** | 17.8 (11.4-26.6) | 24.0 (17.4-28.6) |
|  |  |  |
| **Percentage of Females, median (IQR)** | 49.55 (40.5-53.7) | Not reported^*^ |

^*^1 study of pregnant women, 1 study of adult males only, 2 studies did not report number of females

Table F: Risk of Bias Assessment of Included and Excluded Studies

|  | **Study design and objectives** | **Bias in selection of subjects and constitution of study groups** | **Bias due to withdrawal or loss to follow up (attrition)** | **Information bias regarding the drug safety outcome** | **Other information bias** | **Conflict of interest** | **SUMMARY RISK OF BIAS** |
| --- | --- | --- | --- | --- | --- | --- | --- |
| *Randomised Controlled Trials - Included* | | | | | |  | |
| Ndiaye 2011^5^ |  |  |  |  |  |  |  |
| Ogutu 2014^6^ |  |  |  |  |  |  |  |
| Siqueira 2017^11^ |  |  |  |  |  |  |  |
| WANECAM^8,13^ |  |  |  |  |  |  |  |
| *Randomised Controlled Trials - Excluded* | | | | | | | |
| Mutabingwa 2009^14^ |  |  |  |  |  |  |  |
| Ngouesse 2001^15^ |  |  |  |  |  |  |  |
| Supan 2017^16^ |  |  |  |  |  |  |  |
| *Cohorts - Excluded* | | | | | | | |
| Adjei 2012^17^ |  |  |  |  |  |  |  |

**Legend**

|  | Low |  | Unclear |  | High |
| --- | --- | --- | --- | --- | --- |

As this systematic review was conducted to identify studies for an individual patient data meta-analysis, risk of bias assessment of statistical methods of individual studies was considered not relevant.

## Population Characteristics

Figure D: Histogram of Total Amodiaquine Dose Received


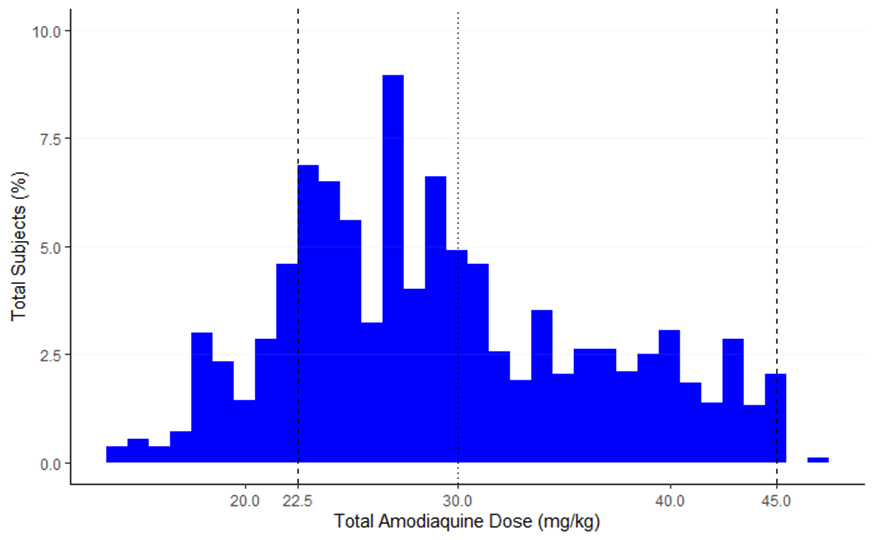


Vertical lines demarcate the WHO-recommended total therapeutic dose (dotted) and range (dashed) for amodiaquine of 30 (22.5-45) mg/kg body weight over 3 days^18^

Figure E: Total Amodiaquine Dose Received by Individual Body Weight


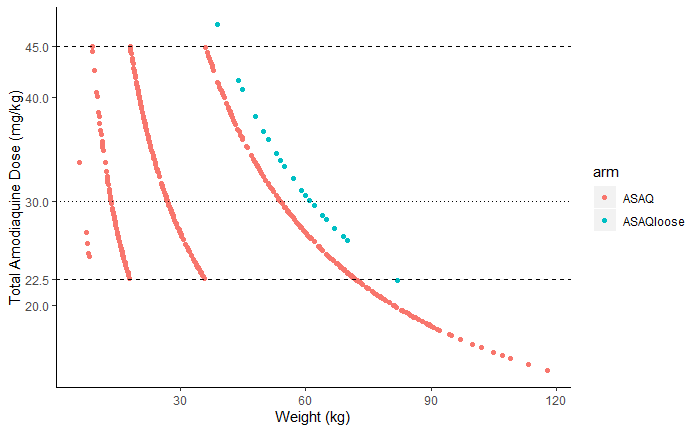


Horizontal lines demarcate the WHO-recommended total therapeutic dose (dotted) and range (dashed) for amodiaquine of 30 (22.5-45) mg/kg body weight over 3 days ^18^

Figure F: Total Non-Amodiaquine Quinoline and Structurally Related Antimalarial Dose Received by Individual Body Weight


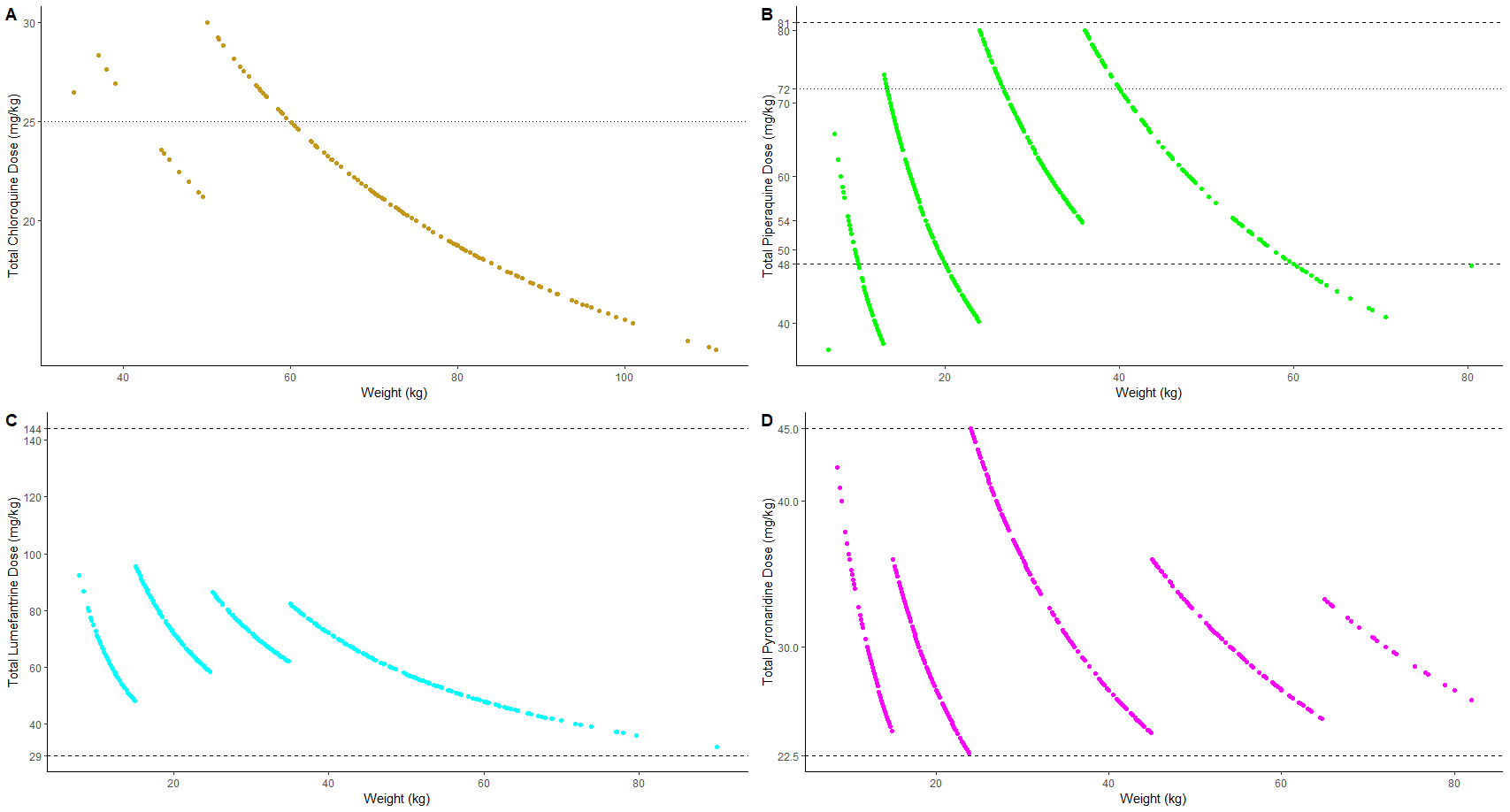


Horizontal lines demarcate the WHO-recommended total therapeutic dose (dotted) and/or range (dashed) over 3 days in units of mg/kg body weight for (A) chloroquine (25), (B) piperaquine (72; 48-81), (C) lumefantrine (29-144), and (D) pyronaridine (22.5-45)

## Corrected QT Interval Analyses

Figure G: Corrected QT Interval and RR Interval Relationships by Correction Method, Treatment Day, and Individual Study


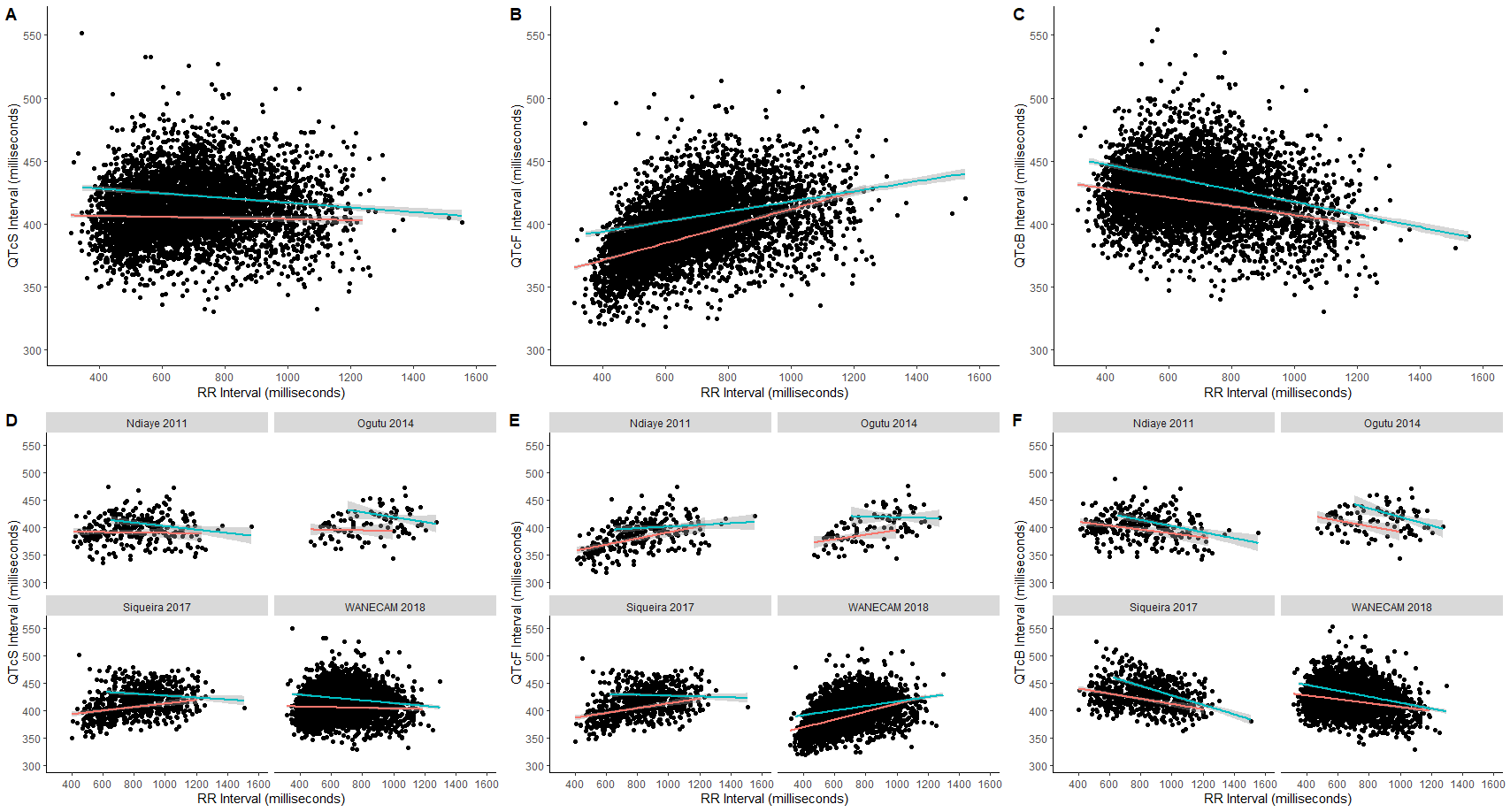


QT intervals adjusted with study-specific ($QTcS=\frac{QT}{{RR}^{\beta_{age}}}$, where$\beta_{age}$ decreased with increasing age), Fridericia ($QTcF = \frac{QT}{\sqrt[3]{RR}}$), and Bazett heart rate corrections ($QTcB = \frac{QT}{\sqrt{RR}}$), and their relationship with RR intervals in the pooled dataset (top panels) and by individual study (bottom panels) with means pre-treatment on day 0 (pink line) and post-treatment on days 2 or 3 (cyan line) with their 95% confidence intervals (shaded area) from linear regression.

Table G: Summary Statistics of Corrected QT Interval Measurements from ECG Interval Analysis by Drug Arm and Time Point

| **Study Reference** | **Ndiaye 2011^5^** | | | **Ogutu 2014^6^** | **Siqueira 2017^7^** | | | **WANECAM 2018^8^** | | | | |
| --- | --- | --- | --- | --- | --- | --- | --- | --- | --- | --- | --- | --- |
| **Drug Arm** | *ASAQ* | *AL* | *p* | *ASAQ* | *ASAQ* | *CQ* | *p* | *ASAQ* | *AL* | *DP* | *PA* | *p* |
| Total dose (mg/kg)^a^ |  |  |  |  |  |  |  |  |  |  |  |  |
| Median (IQR) | 28.2  (25.0-36.1) | 58.8  (50.7-67.3) |  | 29.45  (27.4-31.6) | 22.8  (20.1-26.5) | 20.8  (18.3-23.4) |  | 31.4  (27.0-38.6) | 68.57 (60.5-79.41) | 57.8  (48.0-67.6) | 30.3  (27.0-34.8) |  |
|  |  |  |  |  |  |  |  |  |  |  |  |  |
| **Day 0 (Pre-Dose)** |  |  |  |  |  |  |  |  |  |  |  |  |
| Number of Individuals | 75 | 73 |  | 51 | 177 | 173 |  | 417 | 426 | 716 | 566 |  |
| Corrected QT Interval (ms) |  |  |  |  |  |  |  |  |  |  |  |  |
| Mean QTcS (SD) | 391.7 (20.3) | 390.1 (25.3) | 0.6705^b^ | 394.9 (19.8) | 403.9 (22.2) | 403.5 (23.3) | 0.8607^b^ | 408.6 (19.8) | 406.6 (21.0) | 407.8 (20.5) | 403.8 (20.0) | 0.0017^e^ |
| QTcS>500 | 0 | 0 | 1^c^ | 0 | 1 (0.6%) | 0 | 1^c^ | 0 | 1 (0.2%) | 0 | 0 | 0.3967^c^ |
| QTcF>500 | 0 | 0 | 1^c^ | 0 | 0 | 0 | 1^c^ | 0 | 1 (0.2%) | 0 | 0 | 0.3967^c^ |
| QTcB>500 | 0 | 0 | 1^c^ | 0 | 3 (1.7%) | 2 (1.2%) | 1^c^ | 0 | 1 (0.2%) | 0 | 0 | 0.3967^c^ |
| 480<QTcS≤500 | 0 | 0 | 1^c^ | 0 | 0 | 0 | 1^c^ | 0 | 0 | 0 | 0 | 1^c^ |
| 480<QTcF≤500 | 0 | 0 | 1^c^ | 0 | 1 (0.6%) | 0 | 1^c^ | 0 | 0 | 0 | 0 | 1^c^ |
| 480<QTcB≤500 | 0 | 1 (1.4%) | 0.4932^c^ | 0 | 1 (0.6%) | 3 (1.7%) | 0.3672^c^ | 2 (0.5%) | 1 (0.2%) | 2 (0.3%) | 3 (0.5%) | 0.7553^c^ |
| 450<QTcS≤480 | 0 | 1 (1.4%) | 0.4932^c^ | 0 | 4 (2.3%) | 7 (4.0%) | 0.5149^d^ | 9 (2.2%) | 4 (0.9%) | 14 (2.0%) | 9 (1.6%) | 0.5071^d^ |
| 450<QTcF≤480 | 0 | 1 (1.4%) | 0.4932^c^ | 0 | 4 (2.3%) | 7 (4.0%) | 0.5149^d^ | 2 (0.5%) | 0 | 0 | 1 (0.2%) | 0.1119^c^ |
| 450<QTcB≤480 | 0 | 1 (1.4%) | 0.4932^c^ | 0 | 23 (13.0%) | 19 (11.0%) | 0.6785^d^ | 35 (8.4%) | 25 (5.9%) | 72 (10.1%) | 24 (4.2%) | 0.0005^d^ |
|  |  |  |  |  |  |  |  |  |  |  |  |  |
| **Day 2 or 3 (Post-Dose)** |  |  |  |  |  |  |  |  |  |  |  |  |
| Number of Individuals | 74 | 73 |  | 51 | 175 | 174 |  | 417 | 426 | 716 | 566 |  |
| Corrected QT Interval (ms) |  |  |  |  |  |  |  |  |  |  |  |  |
| Mean QTcS (SD) | 406.7 (25.0) | 400.9 (20.5) | 0.1245^b^ | 418.1 (22.4) | 428.1 (20.3) | 429.6 (20.8) | 0.5024^b^ | 428.1 (21.7) | 415.7 (19.3) | 430.6 (24.3) | 406.3 (19.7) | <0.0001^e^ |
| QTcS>500 | 0 | 0 | 1^c^ | 0 | 0 | 0 | 1^c^ | 2 (0.5%) | 0 | 11 (1.5%) | 0 | 0.0006^c^ |
| QTcF>500 | 0 | 0 | 1^c^ | 0 | 0 | 0 | 1^c^ | 1 (0.2%) | 0 | 4 (0.6%) | 0 | 0.1487^c^ |
| QTcB>500 | 0 | 0 | 1^c^ | 0 | 0 | 1 (0.6%) | 0.4986^c^ | 2 (0.5%) | 0 | 12 (1.7%) | 0 | 0.0003^c^ |
| 480<QTcS≤500 | 0 | 0 | 1^c^ | 0 | 1 (0.6%) | 0 | 1^c^ | 2 (0.5%) | 0 | 14 (2.0%) | 0 | <0.0001^c^ |
| 480<QTcF≤500 | 0 | 0 | 1^c^ | 0 | 0 | 0 | 1^c^ | 2 (0.5%) | 0 | 9 (1.3%) | 0 | 0.0030^c^ |
| 480<QTcB≤500 | 0 | 0 | 1^c^ | 0 | 3 (1.7%) | 10 (5.7%) | 0.0879^d^ | 13 (3.1%) | 2 (0.5%) | 32 (4.5%) | 1 (0.2%) | <0.0001^d^ |
| 450<QTcS≤480 | 3 (4.1%) | 1 (1.4%) | 0.6198^c^ | 5 (9.8%) | 26 (14.9%) | 34 (19.5%) | 0.3089^d^ | 56 (13.4%) | 18 (4.2%) | 106 (14.8%) | 7 (1.2%) | <0.0001^d^ |
| 450<QTcF≤480 | 3 (4.1%) | 1 (1.4%) | 0.6198^c^ | 3 (5.9%) | 24 (13.7%) | 31 (17.8%) | 0.3657^d^ | 16 (3.8%) | 0 | 35 (4.9%) | 0 | <0.0001^d^ |
| 450<QTcB≤480 | 4 (5.4%) | 1 (1.4%) | 0.3664^c^ | 6 (11.8%) | 32 (18.3%) | 45 (25.9%) | 0.1147^d^ | 83 (19.9%) | 41 (9.6%) | 170 (23.7%) | 24 (4.2%) | <0.0001^d^ |
| Change in QTc Interval (ms) |  |  |  |  |  |  |  |  |  |  |  |  |
| ΔQTcS >60 | 2 (2.7%) | 3 (4.1%) | 0.681^c^ | 4 (8.3%)^f^ | 12 (6.9%)^g^ | 6 (3.5%)^g^ | 0.231^d^ | 17 (4.1%) | 2 (0.5%) | 47 (6.6%) | 5 (0.9%) | <0.0001^d^ |
| ΔQTcF >60 | 10 (13.5%) | 5 (6.8%) | 0.2882^d^ | 7 (14.6%)^f^ | 14 (8.1%)^g^ | 11 (6.4%)^g^ | 0.6889^d^ | 47 (11.3%) | 13 (3.1%) | 90 (12.6%) | 13 (2.3%) | <0.0001^d^ |
| ΔQTcB >60 | 1 (1.4%) | 2 (2.7%) | 0.6198^c^ | 1 (2.1%)^f^ | 0 | 1 (0.6%)^g^ | 0.4986^c^ | 13 (3.1%) | 1 (0.2%) | 35 (4.9%) | 2 (0.4%) | <0.0001^d^ |
| 30<ΔQTcS≤60 | 16 (21.6%) | 12 (16.4%) | 0.5551^d^ | 11 (22.9%)^f^ | 53 (30.3%)^g^ | 64 (37.2%)^g^ | 0.2397^d^ | 120 (28.8%) | 66 (15.5%) | 216 (30.2%) | 39 (6.9%) | <0.0001^d^ |
| 30<ΔQTcF≤60 | 27 (36.5%) | 24 (32.9%) | 0.7745^d^ | 21 (43.8%)^f^ | 64 (37.0%)^g^ | 66 (38.4%)^g^ | 0.8784^d^ | 155 (37.2%) | 104 (24.4%) | 271 (37.8%) | 72 (12.7%) | <0.0001^d^ |
| 30<ΔQTcB≤60 | 8 (10.8%) | 7 (9.6%) | 1^d^ | 6 (12.5%)^f^ | 16 (9.2%)^g^ | 27 (15.7%)^g^ | 0.0989^d^ | 82 (19.7%) | 46 (10.8%) | 157 (21.9%) | 23 (4.1%) | <0.0001^d^ |
|  |  |  |  |  |  |  |  |  |  |  |  |  |
| **Day 28 (Late)** |  |  |  |  |  |  |  |  |  |  |  |  |
| Number of Individuals |  |  |  | 49 | 166 | 163 |  |  |  |  |  |  |
| Corrected QT Interval (ms) |  |  |  |  |  |  |  |  |  |  |  |  |
| Mean QTcS (SD) |  |  |  | 394.1 (22.3) | 404.1 (19.3) | 407.2 (20.4) | 0.157^b^ |  |  |  |  |  |
| QTcS>500 |  |  |  | 0 | 0 | 0 | 1^c^ |  |  |  |  |  |
| QTcF>500 |  |  |  | 0 | 0 | 0 | 1^c^ |  |  |  |  |  |
| QTcB>500 |  |  |  | 0 | 0 | 1 (0.6%) | 0.4954^c^ |  |  |  |  |  |
| 480<QTcS≤500 |  |  |  | 0 | 0 | 1 (0.6%) | 0.4954^c^ |  |  |  |  |  |
| 480<QTcF≤500 |  |  |  | 0 | 0 | 0 | 1^c^ |  |  |  |  |  |
| 480<QTcB≤500 |  |  |  | 0 | 1 (0.6%) | 0 | 1^c^ |  |  |  |  |  |
| 450<QTcS≤480 |  |  |  | 0 | 4 (2.4%) | 3 (1.8%) | 1^c^ |  |  |  |  |  |
| 450<QTcF≤480 |  |  |  | 0 | 3 (1.8%) | 4 (2.5%) | 0.7215^c^ |  |  |  |  |  |
| 450<QTcB≤480 |  |  |  | 1 (2.0%) | 7 (4.2%) | 7 (4.3%) | 1^d^ |  |  |  |  |  |
| Change in QTc Interval (ms) |  |  |  |  |  |  |  |  |  |  |  |  |
| ΔQTcS >60 |  |  |  | 0 | 2 (1.2%)^g^ | 0 | 0.4985^c^ |  |  |  |  |  |
| ΔQTcF >60 |  |  |  | 1 (2.2%)^f^ | 3 (1.8%)^g^ | 0 | 0.2477^c^ |  |  |  |  |  |
| ΔQTcB >60 |  |  |  | 0 | 1 (0.6%)^g^ | 0 | 1^c^ |  |  |  |  |  |
| 30<ΔQTcS≤60 |  |  |  | 3 (6.5%)^f^ | 9 (5.5%)^g^ | 10 (6.2%)^g^ | 0.9669^d^ |  |  |  |  |  |
| 30<ΔQTcF≤60 |  |  |  | 7 (15.2%)^f^ | 15 (9.0%)^g^ | 15 (9.3%)^g^ | 1^d^ |  |  |  |  |  |
| 30<ΔQTcB≤60 |  |  |  | 1 (2.2%)^f^ | 1 (0.6%)^g^ | 3 (18.6%)^g^ | 0.3681^c^ |  |  |  |  |  |

IQR = inter-quartile range, SD = standard deviation, ASAQ = artesunate-amodiaquine, AL = artemether-lumefantrine, CQ = chloroquine, DP = dihydroartemisinin-piperaquine, PA = pyronaridine-artesunate, QTcS = QT Interval with study-specific heart rate Correction, QTcF = QT Interval with Fridericia heart rate correction, QTcB = QT Interval with Bazett heart rate correction, ^a^Quinoline and structurally-related drug only, ^b^2-sample t-test with Welch modification, ^c^Fisher’s exact test, ^d^Pearson’s Chi-squared test for independence with Yates’ continuity correction, ^e^Kruskal-Wallis test, ^f^3 participants had missing baseline QT intervals, ^g^2 participants had missing baseline QT intervals

The differences in mean day 2 or 3 (post-dose) QTcS duration between treatment arms in the Ndiaye 2011 and Siqueira 2017 studies respectively comparing artesunate-amodiaquine to artemether-lumefantrine and chloroquine were neither clinically nor statistically significant at the sample sizes of the individual studies. Similarly, the differences between treatment arms in proportions of participants with corrected QT interval absolute values and changes from baseline beyond all thresholds recommended in International Council for Harmonisation of Technical Requirements for Pharmaceuticals for Human Use (ICH) guidelines^19^ were also neither clinically nor statistically significant regardless of the heart rate correction used (Table G).

In the WANECAM 2018 study (n=2125), differences in mean post-dose QTcS intervals among the treatment arms were clinically and statistically significant (*p*<0.0001), with dihydroartemisinin-piperaquine having the highest mean post-dose QTcS (430.6 milliseconds, 95% CI: 428.8-432.4) followed by artesunate-amodiaquine (428.1 milliseconds, 95% CI: 426.0-430.1), then artemether-lumefantrine (415.7 milliseconds, 95% CI: 413.8-417.5) and pyronaridine-artesunate (406.3 milliseconds, 95% CI: 404.7-408.0). However, mean QTcS intervals among the four arms were already significantly different at baseline (*p*=0.0017) even if the differences were small and of borderline clinical significance. Apart from the QTcF>500 millisecond threshold, differences among treatment arms in proportions of participants with corrected QT intervals above ICH thresholds at the post-dose time point were also clinically and statistically significant regardless of heart rate correction used, with the highest proportions in the dihydroartemisinin-piperaquine arm followed by the artesunate-amodiaquine arm, then the artemether-lumefantrine and pyronaridine-artesunate arms, in keeping with mean QTcS findings (Table G).

In the two studies which had late ECG measurements, mean QTcS intervals had returned to baseline levels in all treatment arms by day 28. The differences between treatment arms in proportions of participants with corrected QT interval measurements beyond ICH thresholds were once again neither clinically nor statistically significant regardless of the heart rate correction used (Table G).

Among all included individuals, four participants had uncorrected QT intervals of >500 milliseconds. All occurred post-dose: three (0.4%) among the 716 individuals treated with dihydroartemisinin-piperaquine and one (0.1%) among the 722 artesunate-amodiaquine treated individuals with post-dose QT interval measurements. None of the patients with post-dose QT intervals >500 milliseconds (uncorrected or corrected) experienced any clinical complications.

## Heart Rate Analyses

Table H: Summary Statistics of Heart Rate Measurements from ECG Interval Analyses of Individual Studies by Drug Arm and Time Point

| **Study Reference** | **Ndiaye 2011^5^** | | | **Ogutu 2014^6^** | **Siqueira 2017^7^** | | | **WANECAM 2018^8^** | | | | |
| --- | --- | --- | --- | --- | --- | --- | --- | --- | --- | --- | --- | --- |
| **Drug Arm** | *ASAQ* | *AL* | *p* | *ASAQ* | *ASAQ* | *CQ* | *p* | *ASAQ* | *AL* | *DP* | *PA* | *p* |
| Total dose (mg/kg)^a^ |  |  |  |  |  |  |  |  |  |  |  |  |
| Median (IQR) | 28.2  (25.0-36.1) | 58.8  (50.7-67.3) |  | 29.5  (27.4-31.6) | 22.8  (20.1-26.5) | 20.8  (18.3-23.4) |  | 31.4  (27.0-38.6) | 68.6 (60.5-79.4) | 57.8  (48.0-67.6) | 30.3  (27.0-34.8) |  |
|  |  |  |  |  |  |  |  |  |  |  |  |  |
| **Day 0 (Pre-Dose)** |  |  |  |  |  |  |  |  |  |  |  |  |
| Number of Individuals | 75 | 73 |  | 53 | 177 | 173 |  | 417 | 426 | 716 | 566 |  |
| Heart Rate (beats/minute) |  |  |  |  |  |  |  |  |  |  |  |  |
| Mean (SD) | 94.9 (19.2) | 95.8 (21.9) | 0.7943^b^ | 91.7 (15.7) | 90.3 (21.3) | 88.4 (19.1) | 0.3867^b^ | 109.1 (22.0) | 108.8 (22.0) | 108.9 (21.6) | 106.0 (22.8) | 0.0573^f^ |
| ≤60 or age-equivalent | 2 (2.7%) | 3 (4.1%) | 0.6789^c^ | 1 (1.9%) | 9 (5.1%) | 9 (5.1%) | 1^d^ | 12 (2.9%) | 10 (2.3%) | 21 (2.9%) | 18 (3.2%) | 0.8912^d^ |
| ≤50 or age-equivalent | 1 (1.3%) | 1 (1.4%) | 1^c^ | 0 | 2 (1.1%) | 2 (1.1%) | 1^c^ | 2 (0.5%) | 3 (0.7%) | 4 (0.6%) | 3 (0.5%) | 1^c^ |
|  |  |  |  |  |  |  |  |  |  |  |  |  |
| **Day 2 or 3 (Post-Dose)** |  |  |  |  |  |  |  |  |  |  |  |  |
| Number of Individuals | 74 | 73 |  | 51 | 175 | 174 |  | 417 | 426 | 716 | 566 |  |
| Heart Rate (beats/minute) |  |  |  |  |  |  |  |  |  |  |  |  |
| Mean (SD) | 61.0 (9.2) | 69.9 (10.6) | <0.0001^b^ | 60.4 (7.5) | 61.1 (9.0) | 70.3 (11.3) | <0.0001^b^ | 81.5 (16.6) | 83.3 (16.6) | 85.2 (16.2) | 86.8 (18.0) | <0.0001^f^ |
| ≤60 or age-equivalent | 46 (62.1%) | 21 (28.8%) | <0.0001^d^ | 29 (56.9%) | 96 (54.9%) | 37 (21.3%) | <0.0001^d^ | 112 (26.9%) | 79 (18.5%) | 122 (17.0%) | 65 (11.5%) | <0.0001^d^ |
| ≤50 or age-equivalent | 13 (17.6%) | 2 (2.8%) | 0.0070^d^ | 2 (3.9%) | 14 (8.0%) | 0 | 0.0004^d^ | 21 (5.0%) | 11 (2.6%) | 20 (2.8%) | 10 (1.8%) | 0.02375^d^ |
| Change in Temperature (°C) |  |  |  |  |  |  |  |  |  |  |  |  |
| Median (IQR) | -1.2  (-2.5 to -0.6) | -1.5  (-2.6 to -0.4) | 0.9536^e^ | -1.2  (-2.2 to -0.6) | -1.6  (-2.7 to -0.6) | -1.3  (-2.5 to -0.4) | 0.2653^e^ | -1.4  (-2.2 to -0.6) | -1.3  (-2.3 to -0.4) | -1.3 (-2.1 to -0.5) | -1.2  (-2.3 to -0.3) | 0.4714^g^ |
|  |  |  |  |  |  |  |  |  |  |  |  |  |
| **Day 28 (Late)** |  |  |  |  |  |  |  |  |  |  |  |  |
| Number of Individuals |  |  |  | 49 | 167 | 164 |  |  |  |  |  |  |
| Heart Rate (beats/minute) |  |  |  |  |  |  |  |  |  |  |  |  |
| Mean (SD) |  |  |  | 67.8 (12.9) | 66.5 (12.5) | 69.5 (11.1) | 0.0247^b^ |  |  |  |  |  |
| ≤60 or age-equivalent |  |  |  | 18 (36.7%) | 61 (36.5%) | 35 (21.3%) | 0.0035^d^ |  |  |  |  |  |
| ≤50 or age-equivalent |  |  |  | 3 (6.1%) | 6 (3.6%) | 1 (0.6%) | 0.1211^c^ |  |  |  |  |  |
| Change in Temperature (°C) |  |  |  |  |  |  |  |  |  |  |  |  |
| Median (IQR) |  |  |  | -1.1  (-2.1 to -0.2) | -1.5  (-2.5 to -0.5) | -1.1  (-2.4 to -0.3) | 0.2346^e^ |  |  |  |  |  |

IQR = inter-quartile range, SD = standard deviation, ASAQ = artesunate-amodiaquine, AL = artemether-lumefantrine, CQ = chloroquine, DP = dihydroartemisinin-piperaquine, PA = pyronaridine-artesunate, ^a^Quinoline and structurally-related drug only, ^b^2-sample t-test with Welch modification for unequal variances, ^c^Fisher’s exact test, ^d^Pearson’s Chi-squared test for independence with Yates’ continuity correction, ^e^Wilcoxon rank sum test with continuity correction, ^f^One-way ANOVA test, ^g^Kruskal-Wallis test

In three of the studies, mean heart rates after the final dose of antimalarial treatment on day 2 or 3 (post-dose) in the artesunate-amodiaquine arms were just above 60 beats/minute, the lower limit of the normal range for adolescents and adults (Ndiaye 2011: 61.0 beats/minute, 95% CI: 58.9-63.1; Ogutu 2014: 60.4, 95% CI: 58.3-62.6; Siqueira 2017: 61.1, 95% CI: 59.7-62.4). These means were clinically and statistically significantly lower than in the relevant comparator arms of artemether-lumefantrine (mean: 69.9 beats/minute, 95% CI: 67.4-72.4, *p*<0.0001) and chloroquine (mean: 70.3 beats/minute, 95% CI: 68.7-72.0, *p*<0.0001). All three of these studies had adolescent and adult populations consisting almost entirely of individuals aged ≥12 years (Table H).

In the remaining study (n=2125), which had a median age of <12 years, the differences among the mean post-dose heart rates in the four arms of artesunate-amodiaquine, artemether-lumefantrine, dihydroartemisinin-piperaquine, and pyronaridine-artesunate were statistically significant (*p*<0.0001) with the artesunate-amodiaquine arm having the lowest mean heart rate of 81.5 beats/minute (95% CI: 79.9-83.1). However, none of these means were close to or within the bradycardic range for children aged 2-<12 years who were 72.0% (1529/2125) of this study population and any differences among these means were small and of uncertain clinical significance. Similarly, the differences among the proportions of participants with post-dose heart rates below age-specific thresholds in the four arms were statistically significant (*p*<0.0001 & *p*=0.02375) with the highest proportions in the artesunate-amodiaquine arm although these were all comparable to the proportions seen in non-amodiaquine comparator arms in the other three studies (Table H).

In the two studies which had day 28 (late) ECG measurements, mean heart rates in the artesunate-amodiaquine arms had returned to levels which were clinically comparable to the chloroquine arm (mean: 69.5 beats/minute, 95% CI: 67.7-71.2, *p*=0.0247) by day 28 (Ogutu 2014: 67.8 beats/minute, 95% CI: 64.0-71.5; Siqueira 2017: 66.5, 95% CI: 64.6-68.4). The relative risk of sinus bradycardia after artesunate-amodiaquine remained higher than chloroquine on day 28 (≤60 beats/minute risk ratio: 1.7, 95% CI: 1.2-2.4, p=0.0024; ≤50 beats/minute risk ratio: 5.9, 95% CI: 0.7-48.4, *p*=0.0597).

## Cardiovascular Vital Signs Analyses

Figure H: Pulse Rate and Blood Pressure Measurements after Artesunate-Amodiaquine and Chloroquine for *P. vivax* Malaria
in Brazilian Adults (≥12 years) and Children (<12 years)


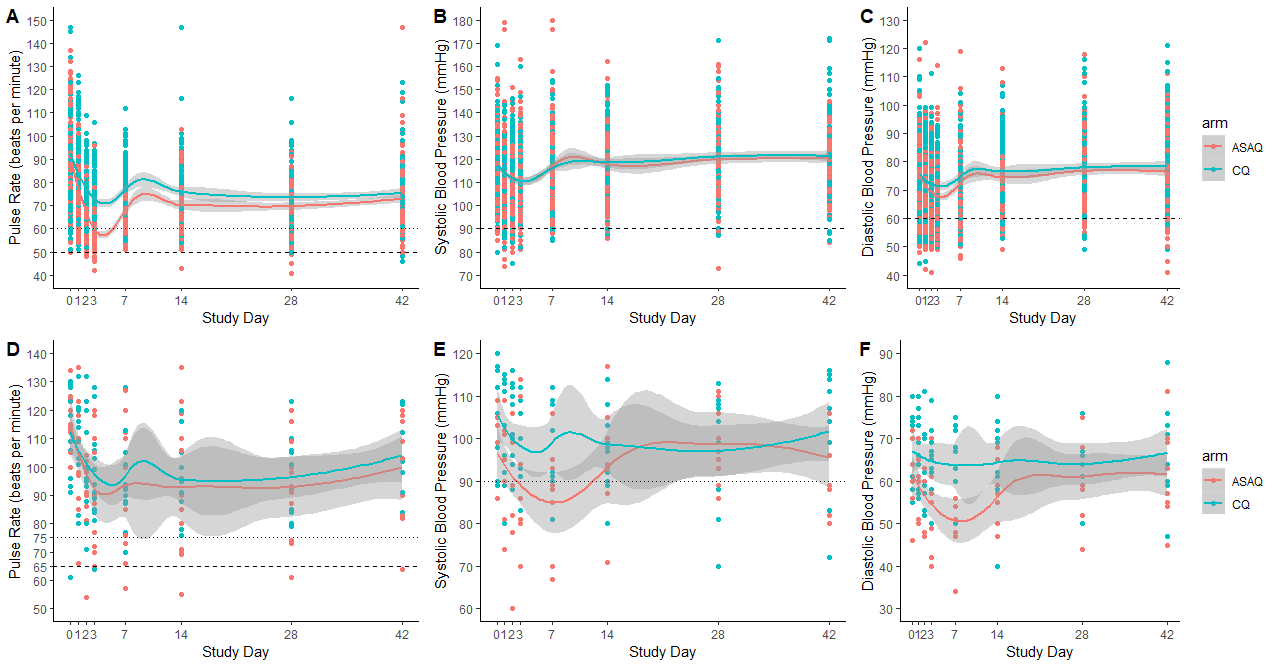


Data from 352 adults who also had ECG monitoring (top panels) and 28 children who did not have ECG monitoring (bottom panels) in a randomised-controlled trial of artesunate-amodiaquine (ASAQ) versus chloroquine (CQ) for treatment of *P. vivax* malaria^7^ with means (smooths) and 95% confidence intervals (shaded area) by treatment arm from LOESS regression. Horizontal lines demarcate lower limit of normal range (dotted) or threshold below which individuals could be symptomatic (dashed).

Figure I: Pulse Rate and Blood Pressure Measurements after Artesunate-Amodiaquine for *P. falciparum* Malaria in Kenyan Adults (≥18 Years)


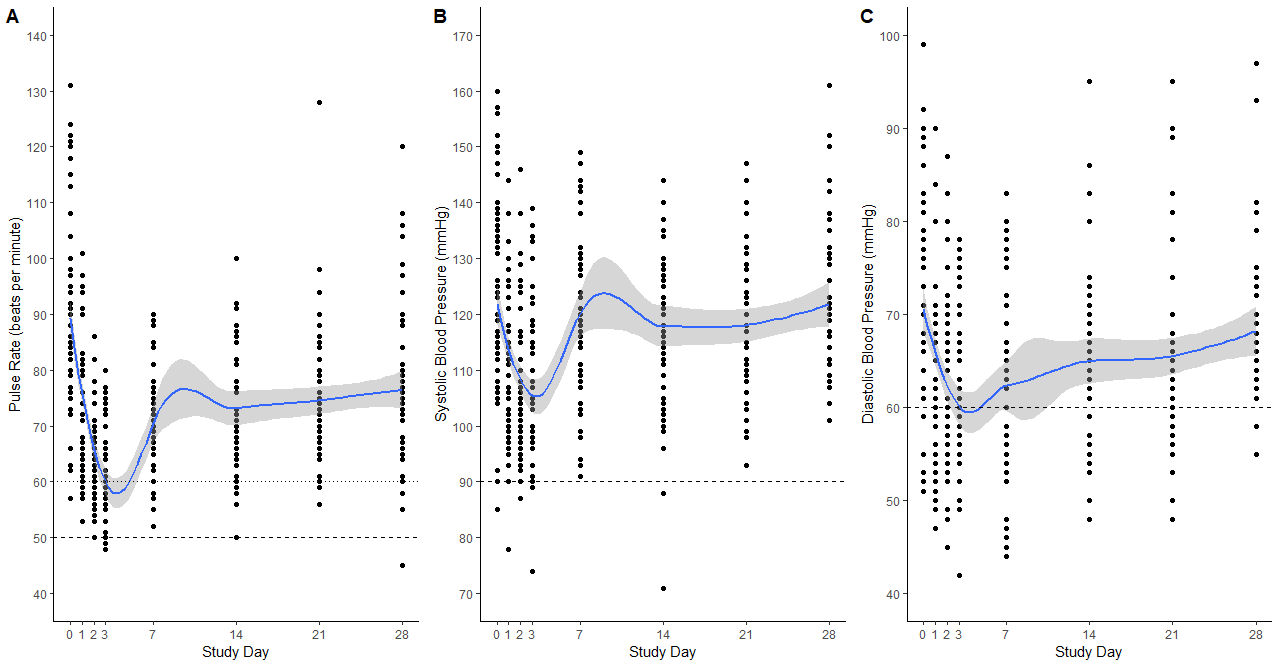


Data from 53 adults who also had ECG monitoring in a randomised-controlled trial of fixed-dose combination versus non-fixed dose combination artesunate-amodiaquine for treatment of *P. falciparum* malaria^6^ with means (smooths) and 95% confidence intervals (shaded area) from LOESS regression. Horizontal lines demarcate lower limit of normal range (dotted) or threshold below which individuals could be symptomatic (dashed).

# References

1. Circle Systems Inc. Stat/Transfer: Data Conversion Software Utility. Seattle, Washington: Circle Systems; 2017.

2. Viskin S, Rosovski U, Sands AJ, et al. Inaccurate electrocardiographic interpretation of long QT: the majority of physicians cannot recognize a long QT when they see one. *Heart Rhythm* 2005; **2**(6): 569-74.

3. Chue AL, Moore RL, Cavey A, et al. Comparability of tympanic and oral mercury thermometers at high ambient temperatures. *BMC Res Notes* 2012; **5**: 356.

4. Lee SJ, Stepniewska K, Anstey N, et al. The relationship between the haemoglobin concentration and the haematocrit in Plasmodium falciparum malaria. *Malar J* 2008; **7**: 149.

5. Ndiaye JL, Faye B, Gueye A, et al. Repeated treatment of recurrent uncomplicated Plasmodium falciparum malaria in Senegal with fixed-dose artesunate plus amodiaquine versus fixed-dose artemether plus lumefantrine: a randomized, open-label trial. *Malar J* 2011; **10**: 237.

6. Ogutu B, Juma E, Obonyo C, et al. Fixed dose artesunate amodiaquine - a phase IIb, randomized comparative trial with non-fixed artesunate amodiaquine. *Malar J* 2014; **13**: 498.

7. Siqueira AM, Alencar AC, Melo GC, et al. Fixed-Dose Artesunate-Amodiaquine Combination vs Chloroquine for Treatment of Uncomplicated Blood Stage P. vivax Infection in the Brazilian Amazon: An Open-Label Randomized, Controlled Trial. *Clin Infect Dis* 2016.

8. West African Network for Clinical Trials of Antimalarial D. Pyronaridine-artesunate or dihydroartemisinin-piperaquine versus current first-line therapies for repeated treatment of uncomplicated malaria: a randomised, multicentre, open-label, longitudinal, controlled, phase 3b/4 trial. *Lancet* 2018; **391**(10128): 1378-90.

9. Fleming S, Thompson M, Stevens R, et al. Normal ranges of heart rate and respiratory rate in children from birth to 18 years of age: a systematic review of observational studies. *Lancet* 2011; **377**(9770): 1011-8.

10. Textor J, van der Zander B, Gilthorpe MS, Liskiewicz M, Ellison GT. Robust causal inference using directed acyclic graphs: the R package 'dagitty'. *Int J Epidemiol* 2016; **45**(6): 1887-94.

11. Siqueira AM, Alencar AC, Melo GC, et al. Fixed-Dose Artesunate–Amodiaquine Combination vs Chloroquine for Treatment of Uncomplicated Blood Stage P. vivax Infection in the Brazilian Amazon: An Open-Label Randomized, Controlled Trial. *Clinical Infectious Diseases: An Official Publication of the Infectious Diseases Society of America* 2017; **64**(2): 166-74.

12. Funck-Brentano C, Ouologuem N, Duparc S, et al. Evaluation of the effects on the QT-interval of 4 artemisinin-based combination therapies with a correction-free and heart rate-free method. *Sci Rep* 2019; **9**(1): 883.

13. Sagara I, Beavogui AH, Zongo I, et al. Safety and efficacy of re-treatments with pyronaridine-artesunate in African patients with malaria: a substudy of the WANECAM randomised trial. *The Lancet Infectious Diseases* 2016; **16**(2): 189-98.

14. Mutabingwa TK, Muze K, Ord R, et al. Randomized trial of artesunate+amodiaquine, sulfadoxine-pyrimethamine+amodiaquine, chlorproguanal-dapsone and SP for malaria in pregnancy in Tanzania. *PLoS One* 2009; **4**(4): e5138.

15. Ngouesse B, Basco LK, Ringwald P, Keundjian A, Blackett KN. Cardiac effects of amodiaquine and sulfadoxine-pyrimethamine in malaria-infected African patients. *Am J Trop Med Hyg* 2001; **65**(6): 711-6.

16. Supan C, Mombo-Ngoma G, Kombila M, et al. Phase 2a, Open-Label, 4-Escalating-Dose, Randomized Multicenter Study Evaluating the Safety and Activity of Ferroquine (SSR97193) Plus Artesunate, versus Amodiaquine Plus Artesunate, in African Adult Men with Uncomplicated Plasmodium falciparum Malaria. *Am J Trop Med Hyg* 2017; **97**(2): 514-25.

17. Adjei GO, Oduro-Boatey C, Rodrigues OP, et al. Electrocardiographic study in Ghanaian children with uncomplicated malaria, treated with artesunate-amodiaquine or artemether-lumefantrine. *Malar J* 2012; **11**: 420.

18. World Health Organization. Guidelines for the Treatment of Malaria. 3rd ed. Geneva, Switzerland; 2015.

19. ICH Harmonised Tripartite Guideline E14. The Clinical Evaluation of QT/QTc Interval Prolongation and Proarrhythmic Potential for Non-Antiarrhythmic Drugs, 2005.
